# Supplementary material for: A sensitive method for profiling cell-type-specific secretomes of rare cells from complex tissues
Source: Cell Rep Methods. 2026 Aug 14;6(10):101552. doi: 10.1016/j.crmeth.2026.101552 (PMC13721748; doi:10.1016/j.crmeth.2026.101552)
Supplement: Supplementary file 1 [file mmc1.pdf]

Cell Reports Methods, Volume *6*

Supplemental information

A sensitive method for profiling cell-type-specific secretomes of rare cells from complex tissues

Gergana Shipkovenska, Xuchao Lyu, Manalee Vishnu Surve, Joao A. Paulo, Edward Chen, Vanshika Panchal, Brian Lin, Steven P. Gygi, Wei Wei, Jonathan Z. Long, and Jayaraj Rajagopal

Figure S1. Strategy for inducing cell type-specific ERTurboID. Related to Figure 1.

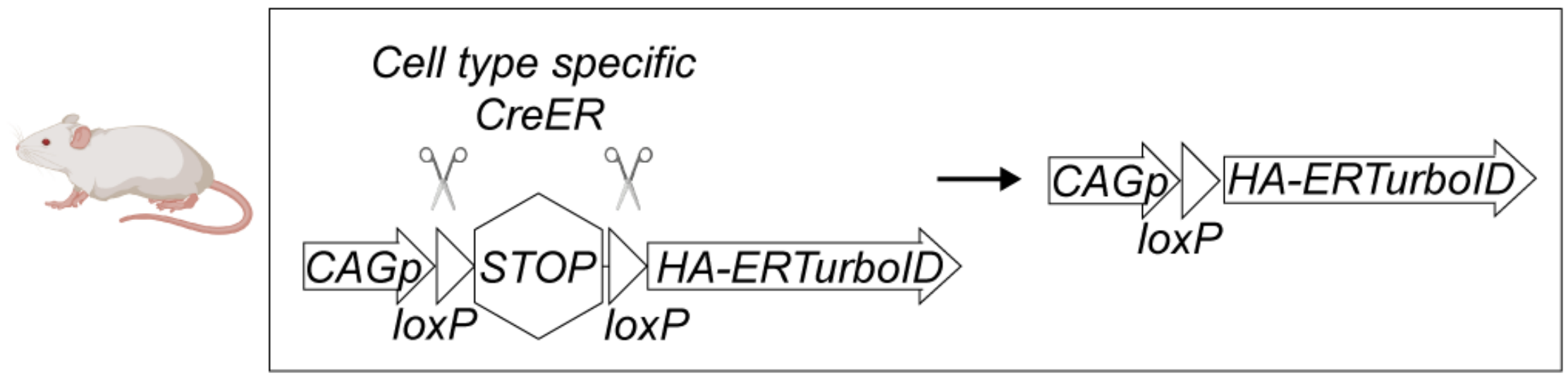

Figure S2. Profiling the secretome of hepatocytes in mice *in vivo*. Related to Figure 2.

A. Schematic of experimental design for profiling the secreted proteome of hepatocytes from intact liver. B. Gene set enrichment analysis of the hepatocyte secretome enriches for terms associated with hepatocyte cell fate. C. GO term analysis of the hepatocyte secretome enriches for terms associated with intracellular compartments involved in protein secretion. P-values were calculated from Fisher exact test. See also Tables S2, S4, S6 and S15.

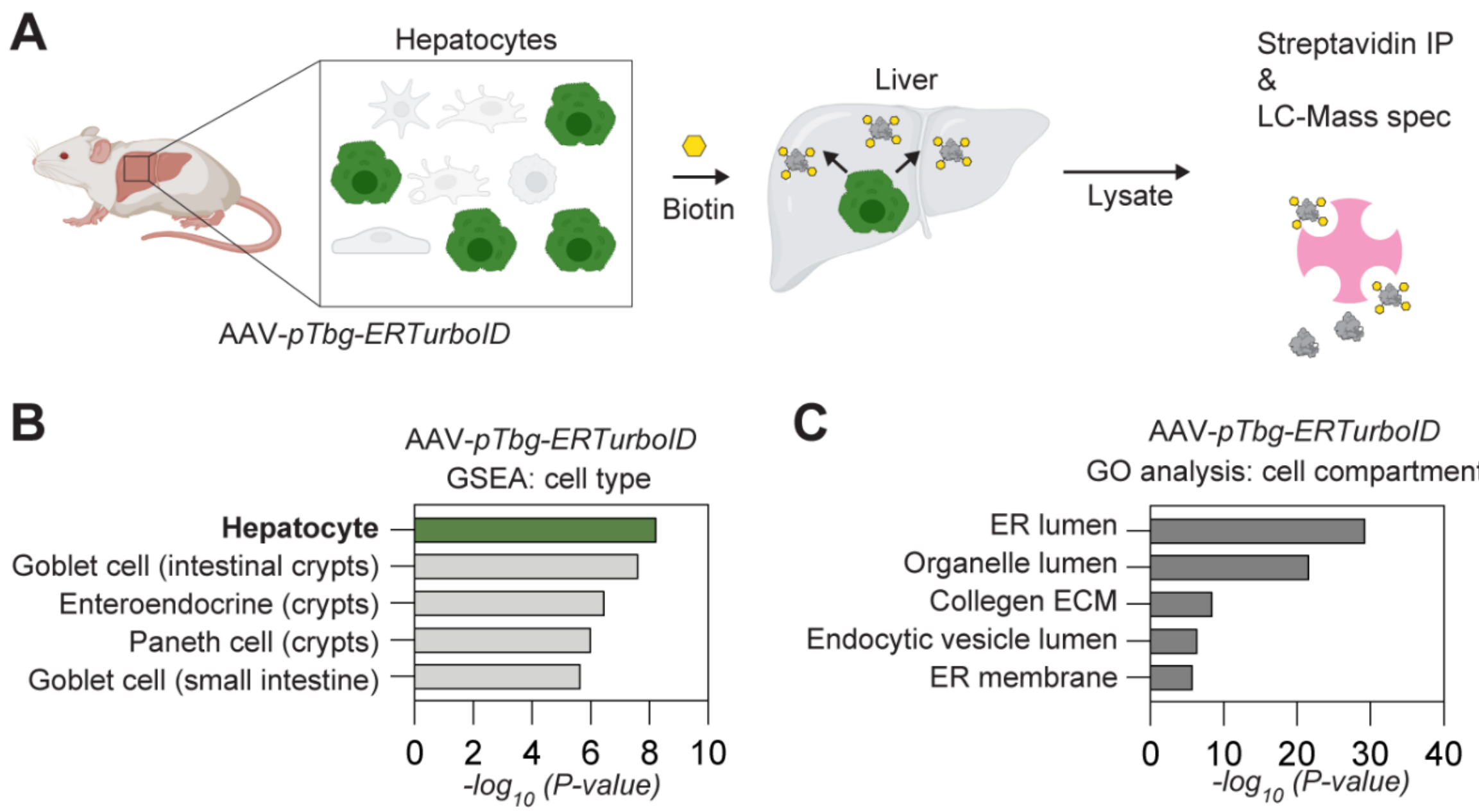

Figure S3. Experimental validation of the *Sox8-CreER* driver. Related to Figure 3.

A. Experimental setup for inducing ERTurboID expression in M cells in air-liquid interface (ALI) cultures. B. Immunofluorescence for M cell markers SOX8 and CCL9 and HA-tagged ERTurboID in fixed ALI cultures of murine airway epithelia. Abbreviations: 4OHT (4 hydroxytamoxifen), RANKL (RANK ligand). C. Quantification of SOX8+ ERTurboID+ double positive cells as proportion of total SOX8 positive cells. Error bars represent standard deviation of two independent cell lines with 6 technical replicates each. D. Quantification of CCL9+ ERTurboID+ double positive cells as proportion of total CCL9 positive cells. Error bars represent standard deviation of two independent cell lines with 6 technical replicates each. E. Quantification of SOX8+ ERTurboID+ or CCL9+ ERTurboID+ double positive cells, as proportion of total ERTurboID positive cells. Error bars represent standard deviation of two independent cell lines with 6 technical replicates each.

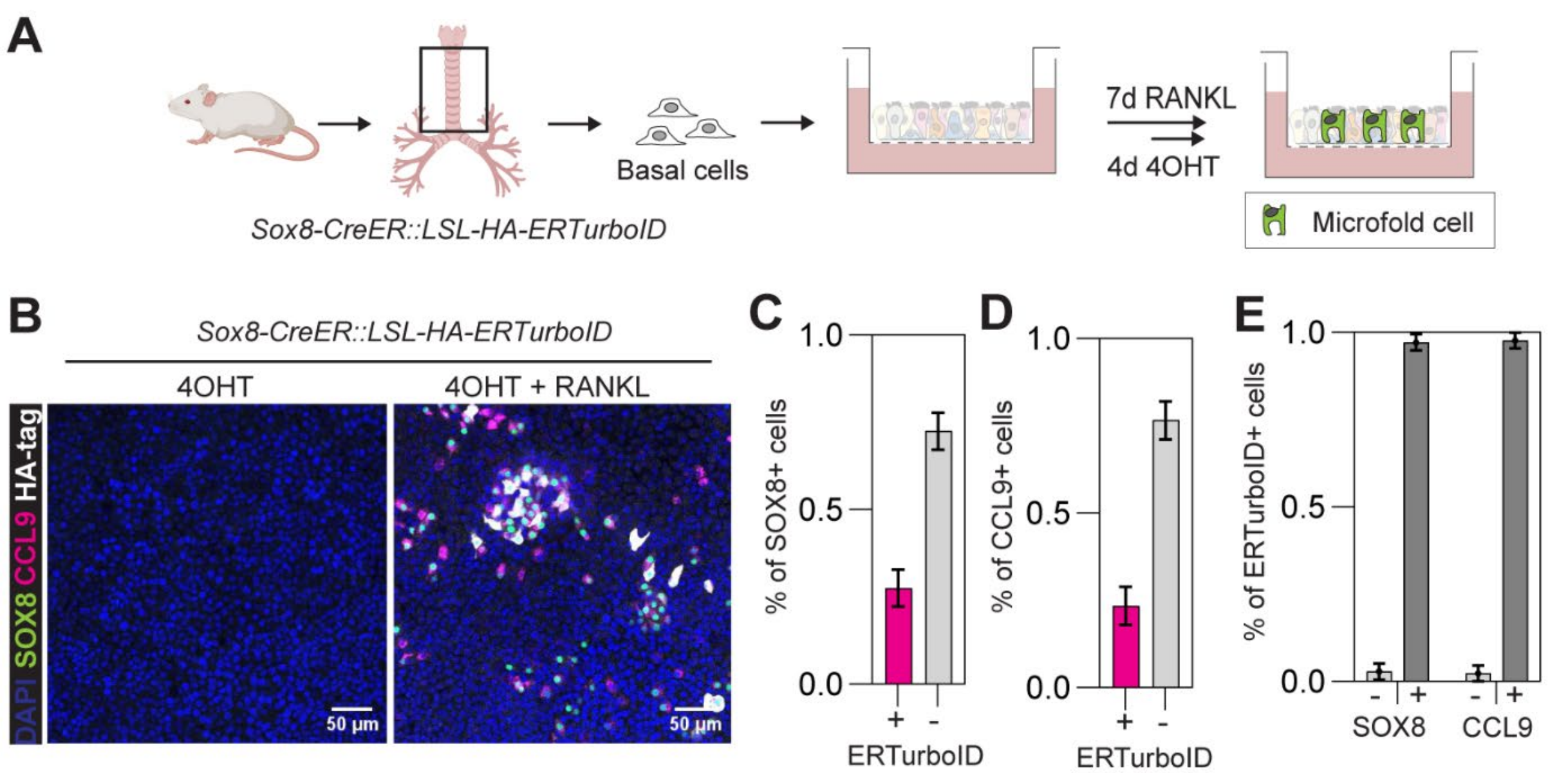

Figure S4. A. Schematic of M cell proteins found in specific compartments of the secretory pathway. B. Immunofluorescence for SOX8, an M cell marker, and TNFRSF11B, which was identified in the M cell secretome. Arrows denote double positive cells. C. Immunofluorescence for SOX8 and VCAM1, which was identified in the M cell secretome. Arrows denote double positive cells. Inset shows magnification of one double positive cell and an orthogonal view along the z-axis to illustrate the luminal-basolateral distribution of VCAM1. Z-sections representing the basolateral and luminal membranes. Related to Figure 4.

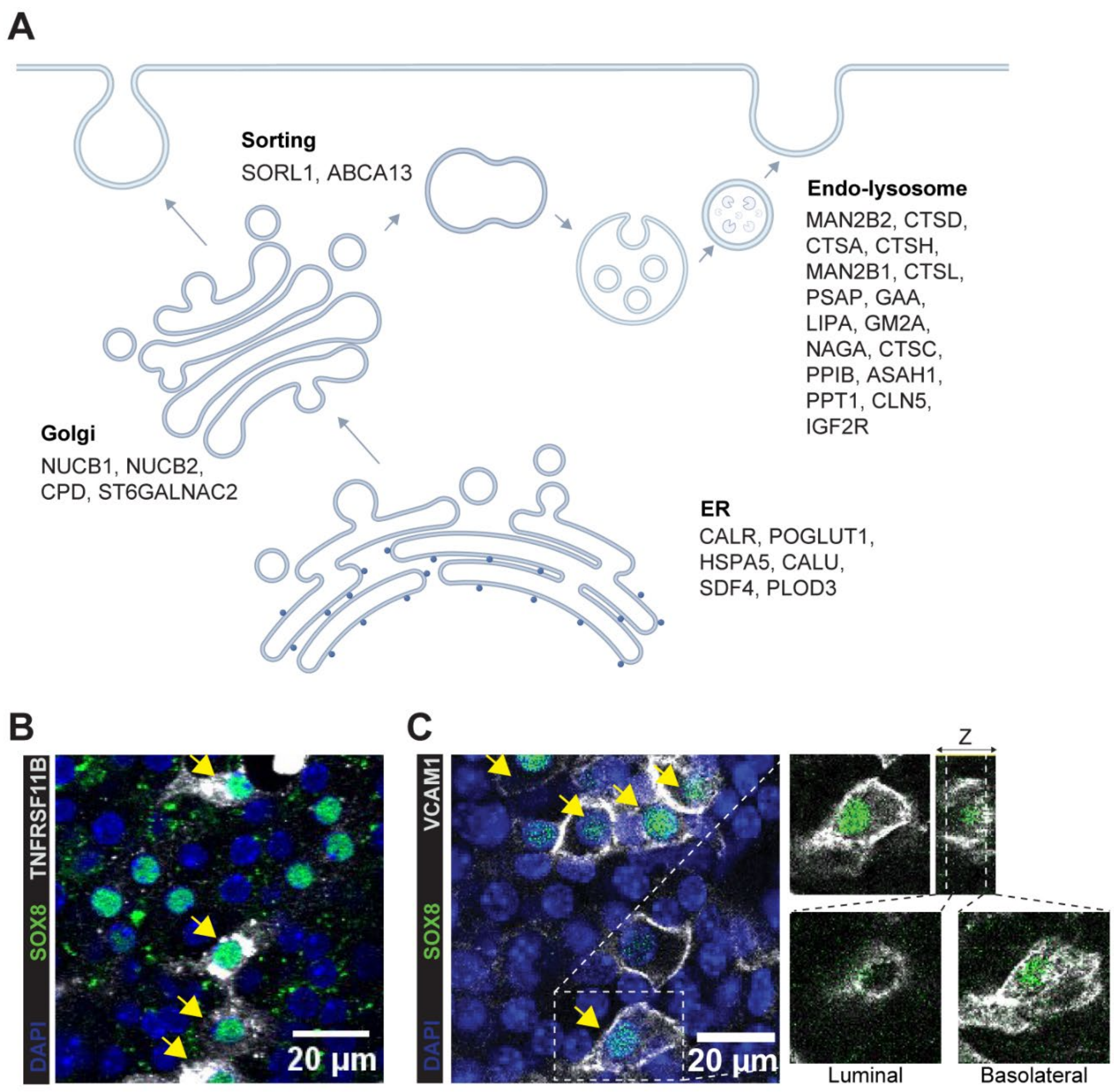

Table S1. List of secreted proteins from Kupffer cells. Related to Figure 2.

Gene	Mean Enrichment Score	Peptides
H1-3	2.068192476	3
Serpina1b	1.565857391	3
TurboID	1.51669736	6
Try4	1.484649371	4
H3c2	1.463571274	5
Cd5l	1.303351435	12
Mia3	1.284448056	14
C1qb	1.185796502	6
Serping1	1.176042236	6
H4c1	1.171030615	5
Apoc1	1.170455665	4
Mrc1	1.100740506	6
Mia2	1.060287106	5
Ganab	1.059353229	7
Ica	1.034277933	9
Apoh	1.028168784	7
Qprt	1.011581575	3
Plg	1.006465476	8
C8g	0.963258798	5
Tpm3	0.961151878	4
H6pd	0.958492919	11
Igkv1-135	0.956383102	4
F5	0.921261736	3
Cfi	0.915050667	4
Cfhr4	0.889943217	5
Ighv3-6	0.886000907	4
Ppia	0.837271137	3
Sdha	0.79476108	3
Ighv8-8	0.761474528	3
Lrp1	0.748487352	14
Aldh6a1	0.708086037	20
Clec4f	0.700801766	4
Rps27a	0.676259129	5
Itih4	0.671665069	14
Ces2e	0.653480864	4
Mpst	0.652614157	3
P4hb	0.642187877	11
Masp2	0.627151401	3
Atp1b1	0.615840486	3
Krt2	0.615290469	5
Atp6v1a	0.613048692	4
Fmo1	0.601157964	5
Dld	0.592919017	5
Pls3	0.561500787	3
Cct2	0.555477939	3
Gapdh	0.551419286	17
Rpl7a	0.546271996	4

Uggt1	0.538522635	9
Atp5pb	0.531088418	5
Nucb1	0.511278665	8
C1ra	0.508431638	5
Ubr4	0.507338881	6

Table S2. List of secreted proteins from hepatocytes. Related to Figure S2.

Gene	Mean Enrichment Score	Peptides
TurboID	4.540224029	25
Creld2	7.094378904	8
Ces3a	5.634971874	22
Ces2e	4.991047056	21
Txn	4.928261864	3
Slco1a1	4.826677035	20
Lypla2	4.766687135	4
Ces1f	4.690017277	35
Tmed10	4.678655188	6
Ces2a	4.660349401	45
Ces1e	4.597166158	15
Cnpy3	4.526766173	6
Ykt6	4.263039871	3
Manf	4.251526506	5
Hspa13	4.012684562	5
Nenf	3.998156244	3
Tmed9	3.988394564	3
Ces2c	3.972517314	17
Creld1	3.900925356	3
Por	3.826866328	12
Prkcsh	3.781182743	14
Slco2b1	3.771868367	5
Pdia3	3.768196921	22
Mesd	3.703087352	12
Pdia4	3.639694069	28
P4hb	3.566127748	43
Cxadr	3.505245909	6
Dnajc3	3.393265269	11
Slc38a3	3.340462264	4
Ero1a	3.307925152	9
Ugt2b36	3.226228089	8
Ces3b	3.193518581	5
Cr1l	3.149322938	3
Hyou1	3.12804866	40
Ero1b	3.09001156	3
Acot2	3.059138254	3
Pcyox1	3.045741912	6
Slco1b2	3.036535014	7
Ppib	2.990278626	3
Ces1d	2.970027545	12
Erp29	2.928201359	4
Lrpap1	2.894345246	11
Ganab	2.888157019	33
Calr	2.870841901	17
Aadac	2.869729728	13
Cnpy4	2.858950646	3
Cyp7b1	2.858768736	3
Mttp	2.853919375	45
Csad	2.810608242	5
Bcap31	2.721963079	7
Ctsh	2.688414569	3

Ces2g	2.668470171	3
Erp44	2.662479779	3
Itgb1	2.649981422	5
Btd	2.623323764	5
Txndc12	2.557570548	3
Ugt2b5	2.518585275	17
H6pd	2.478622775	32
H2-K1	2.467432829	4
Slc9a3r1	2.464274928	3
Selenof	2.451011824	3
Apmap	2.445001551	3
Asah1	2.436091587	3
Lman2	2.431913291	5
Pcyt2	2.420254307	5
Mia3	2.412188929	12
Igfbp2	2.367977519	5
Ces1	2.358982547	10
Coro7	2.342444842	3
Uggt1	2.326399996	27
Bsg	2.233748057	3
Ambp	2.206856355	7
Pofut1	2.133254749	3
Mlec	2.07047889	11
Nucb1	2.008854627	19
F13b	1.967998771	12
Asgr1	1.966187494	6
Col18a1	1.951769465	6
Pebp1	1.868210752	3
Hsd11b1	1.769488656	5
Ctsl	1.763337245	5
Arg1	1.753513148	8
Canx	1.725916678	5
Apoe	1.713968485	10
Timm44	1.64672072	6
Stbd1	1.54164146	3
Lcat	1.536837716	5
Txndc5	1.530036301	4
Dlat	1.453225092	9
Vim	1.44718961	4
Cct8	1.442158416	6
AI182371	1.390925416	10
Ctsd	1.360744343	4
Hrg	1.345929538	12
Man1a1	1.279202792	4
Calu	1.24892885	4
Serpina1b	1.220059127	4
Ca3	1.17151809	11
Klk1b9	1.161944814	6
F2	1.161269476	12
Ugt2b35	1.15065082	10
Cnpy2	1.148333722	3
Alb	1.133199789	59
Hp	1.086918332	7

Apoa4	1.067843856	22
Ces1b	1.065850769	8
Aldh1a1	1.044162996	10
Azgp1	1.029472651	12
Fabp1	1.023229388	3
Prdx6	1.019571399	6
Spcs2	0.960715802	3
Gc	0.916915775	27
Fcn1	0.907971074	3
Serpina3g	0.893332683	11
Abcb11	0.888767941	12
H2-K1	0.879379037	3
Ugt3a2	0.874191579	7
Tkt	0.860572417	3
H4c1	0.839770408	4
Serpina1d	0.837376516	6
Park7	0.805412837	4
Gm20547	0.780716951	17
Fetub	0.747542439	13
Clu	0.715896792	26
Selenbp2	0.695486197	10
Comt	0.688325475	10
Lman1	0.686477808	12
Gclc	0.682851774	4
H2-Q10	0.668616571	6
Ndrg2	0.667199631	4
Serpina1e	0.647953397	3
Serpina3m	0.647772939	35
Gstm1	0.641809707	6
Cfi	0.62987691	14
Apoa1	0.606637175	34
Serpina1a	0.603243317	23
Ctsz	0.599820605	5
C3	0.596452046	70
Kng2	0.587565469	9
F12	0.560299038	4
Serpinf1	0.558713236	7
Tf	0.546796264	74
Cfh	0.542415411	50
Cyb5a	0.540885013	3
C8a	0.537994982	23
Hpx	0.533540971	18
Entpd5	0.525698522	16
C1sa	0.504311858	13

Table S3. Gene Set Enrichment Analysis of the Kupffer cell secretome for cell type-specific markers using the CellMarker 2024 Database (mouse). Related to Figure 2.

**term**	**p-value**	**q-value**	**overlap_genes**
Kupffer Cell Liver Mouse	0.000004	0.000638	CD5L, MRC1, CLEC4F
Macrophage Peritoneal Cavity Mouse	0.000099	0.003642	CD5L, CLEC4F
Macrophage Bone Marrow Mouse	0.000102	0.003642	C1QB, MRC1, F5
Transit Amplifying (TA) Cell Intestinal Crypt Mouse	0.000342	0.010135	P4HB, RPS27A, PPIA, GAPDH
Macrophage Liver Mouse	0.000591	0.013159	C1QB, CLEC4F

Table S4. Gene Set Enrichment Analysis of hepatocyte secretome for cell type-specific markers using the CellMarker 2024 Database (mouse). Related to Figure S2.

**term**	**p-value**	**q-value**	**overlap_genes**
Hepatocyte Liver Mouse	5.680601e-09	1.013987e-06	GSTM1, AMBP, ARG1, APOA1, APOE, CYP7B1, ASGR1
Goblet Cell Intestinal Crypt Mouse	2.425794e-08	2.886694e-06	PDIA3, CYB5A, TMED9, GSTM1, MTTP, SELENOF, APOA1, APOA4, MANF, FABP1, LMAN2, BSG, ALDH1A1, TXNDC5
Enteroendocrine Cell Intestinal Crypt Mouse	3.387809e-07	3.023619e-05	FABP1, CYB5A, GSTM1, IGFBP2, MTTP, ALDH1A1, APOA1, APOA4, VIM
Paneth Cell Intestinal Crypt Mouse	9.642755e-07	6.884927e-05	PDIA3, FABP1, GSTM1, BSG, MTTP, ALDH1A1, APOA1, APOA4, P4HB, COMT, NDRG2, TXNDC5
Goblet Cell Small Intestine Mouse	2.216027e-06	1.318536e-04	PDIA3, TMED9, SELENOF, CALR, CRELD2, TXNDC5, MANF

Table S5. Gene Set Enrichment Analysis of Kupffer cell secretome for markers of subcellular compartments using the GO Cellular Component 2025 Database. Related to Figure 2.

**term**	**p-value**	**q-value**	**overlap_genes**
Endoplasmic Reticulum Lumen (GO:0005788)	5.178072e-08	0.000002	GANAB, H6PD, FMO1, SERPING1, NUCB1, MIA3, P4HB, UGGT1, F5
Intracellular Organelle Lumen (GO:0070013)	5.330122e-08	0.000002	MPST, H6PD, ATP5PB, FMO1, MIA3, SDHA, F5, ALDH6A1, GANAB, SERPING1, NUCB1, P4HB, PPIA, UGGT1
Secretory Granule Lumen (GO:0034774)	1.672427e-05	0.000446	ITIH4, CCT2, APOH, SERPING1, PLG, PPIA, F5
Chylomicron (GO:0042627)	2.359143e-04	0.004718	APOH, APOC1
Vesicle (GO:0031982)	3.222724e-04	0.005156	ATP1B1, RPS27A, PPIA, GAPDH, F5

Table S6. Gene Set Enrichment Analysis of hepatocyte secretome for markers of subcellular compartments using the GO Cellular Component 2025 Database. Related to Figure S2.

term	p-value	q-value	overlap_genes
Endoplasmic Reticulum Lumen (GO:0005788)	5.408981e-30	6.653046e-28	ERO1A, LRPAP1, COL18A1, ERO1B, H6PD, PRKCSH, MTTP, CTSZ, SELENOF, TXNDC12, MIA3, C3, GANAB, APOE, UGGT1, TXNDC5, PDIA3, APOA1, APOA4, CNPY3, MANF, DNAJC3, ERP44, TF, CANX, ERP29, CALU, NUCB1, HYOU1, CALR, P4HB, PPIB, CES1
Intracellular Organelle Lumen (GO:0070013)	2.225699e-22	1.368805e-20	FCN1, ERO1A, COL18A1, ERO1B, ASAH1, H6PD, PRKCSH, MTTP, CTSZ, SELENOF, TXNDC12, MIA3, C3, HPX, GANAB, CTSL, CTSH, APOE, DLAT, CCT8, CTSD, UGGT1, TXNDC5, PDIA3, APOA1, TIMM44, APOA4, CNPY3, MANF, DNAJC3, ERP44, TF, CANX, ACOT2, ERP29, CALU, NUCB1, HYOU1, CALR, P4HB, PPIB, CES1
Collagen-Containing Extracellular Matrix (GO:0062023)	3.423325e-09	1.403563e-07	FCN1, COL18A1, AMBP, F12, SERPINF1, CTSZ, APOA1, APOA4, CLU, HPX, LMAN1, AZGP1, CTSL, CTSH, APOE, CRELD1, CTSD
Endocytic Vesicle Lumen (GO:0071682)	3.707619e-07	1.140093e-05	HPX, CTSL, HYOU1, APOE, CALR
Endoplasmic Reticulum Membrane (GO:0005789)	1.655017e-06	4.071341e-05	BCAP31, ERO1A, CYB5A, ERO1B, TMED9, PCYT2, TMED10, PRKCSH, MIA3, CYP7B1, HSD11B1, ERP44, LMAN1, SPCS2, LMAN2, BSG, CANX, CALU, MLEC, CALR, TKT

Table S7. Gene Set Enrichment Analysis of Kupffer cell secretome for functional pathways using the WikiPathways 2024 Mouse Database. Related to Figure 2.

term	p-value	q-value	overlap_genes
Complement Activation Classical Pathway WP200	0.000879	0.007743	C1QB, C1RA
Statin Pathway WP1	0.001102	0.007743	LRP1, APOC1
Blood Clotting Cascade WP460	0.001223	0.007743	PLG, F5
Amino Acid Metabolism WP662	0.001939	0.009208	MPST, SDHA, DLD
TCA Cycle WP434	0.002938	0.011164	SDHA, DLD

Table S8. Luminally secreted proteins from airway M cells. Related to Figure 3.

Gene	log2 +RANKL/ -RANKL	log2 +RANKL/ +Inhibitor	Unique Peptides	Razor Peptides
Il4i1	3.715226	2.019068	0	161
Mmp7	3.633393	0.729716	0	89
Lama3	3.522197	2.729716	0	68
Lgals3bp	3.522197	3.522197	33	11
Fcgbp	3.133393	3.133393	0	31
Man2b1	2.98864	2.98864	29	0
Man2b2	2.696159	2.696159	10	25
Psap	2.696159	1.696159	0	27
Serpina10	2.696159	2.696159	0	28
Matn2	2.5	2.5	0	29
Ctsh	2.453445	2.453445	0	39
Ecm1	2.453445	1.453445	0	3
Ecm1	2.453445	1.453445	0	68
Lamc2	2.453445	2.453445	7	32
Ctsd	2.437235	1.229716	0	60
Poglut1	2.403677	1.903677	40	0
Fcamr	2.321928	2.321928	0	35
Tgfb2	2.321928	2.321928	6	13
H2-Q4	2.292481	1.292481	0	6
Mfge8	2.289344	0.957861	0	232
Cd14	2.239024	3.642701	75	0
St6galnac2	2.160964	0.868483	39	0
Vcam1	2.160964	2.160964	0	40
Thbs1	2.084963	1.084963	0	18
Icam1	2	1.5	0	19
Ovos	2	2.792481	21	13
Gm20547	1.993964	2.447409	0	102
Sdf4	1.792481	1.292481	5	13
Tnc	1.792481	1.792481	0	12
Tnfrsf11b	1.792481	1.792481	24	0
Hp	1.584963	2.584963	27	0
Clu	1.558894	1.39793	81	129
Pigr	1.549768	1.631517	87	8
Pam	1.292481	1.292481	0	38
Sftpd	0.903677	1.374231	65	0
Mup8	0.792481	2.292481	0	2
Ltf	0.641072	1.53103	139	95
Msln	0.614409	0.710732	0	222

Table S9. Basolaterally secreted proteins from airway M cells. Related to Figure 3.

Gene	log2 +RANKL/ -RANKL	log2 +RANKL/ +Inhibitor	Unique Peptides	Razor Peptides
Nucb1	4.554262	1.094831	0	170
Mfge8	4.518087	4.518087	0	232
App	4.39068	3.39068	0	55
Serpinb1a	4.245927	3.745927	53	65
Aplp2	4.169925	4.169925	0	32
Msln	4.054262	1.163582	0	222
H2-D1	4	3.5	0	64
Calr	3.98864	2.696159	193	0
Vcam1	3.903677	3.903677	0	40
Il4i1	3.877444	2.292481	0	161
Lama3	3.836213	3.836213	0	68
H2-K1	3.753897	3.253897	0	58
Bpifb1	3.745927	1.049768	148	0
Poglut1	3.70044	2.70044	40	0
Man2b2	3.696159	3.696159	10	25
Mmp7	3.696159	1.535195	0	89
Chadl	3.642701	3.642701	8	15
Gm20547	3.642701	3.642701	0	102
Cln5	3.633393	3.633393	26	0
Sorl1	3.633393	3.633393	29	0
Igf2r	3.564642	3.564642	33	0
Ptprk	3.522197	3.522197	0	21
Ctsd	3.511184	2.011184	0	60
Dsg2	3.453445	3.453445	92	47
Met	3.447409	3.447409	0	26
Lgals3bp	3.39068	3.39068	33	11
Plxnb2	3.39068	3.39068	52	0
Tnfrsf11b	3.39068	3.39068	24	0
Cd14	3.377444	3.377444	75	0
Matn2	3.377444	3.377444	0	29
Spint1	3.321928	3.321928	23	0
Ltf	3.253897	1.85022	139	95
Ptprz1	3.245927	3.245927	0	17
Pigr	3.20044	3.20044	87	8
Serpina10	3.169925	3.169925	0	28
Ctsa	3.160964	3.160964	0	27
Ctsh	3.084963	3.084963	0	39
Lamc2	3.084963	3.084963	7	32
Fcgbp	3.064642	3.064642	0	31
Man2b1	3.064642	3.064642	29	0
H2-Q4	3	2.5	0	6
Ldlr	3	3	0	17
Fat1	2.98864	2.98864	0	20
Lamb3	2.953445	2.953445	29	0
Nucb2	2.903677	0.903677	0	75
Psap	2.877444	2.877444	0	27
Cd74	2.807355	2.807355	1	34
Ctsl	2.807355	1.514874	1	34
L1cam	2.807355	2.807355	0	14

Sftpd	2.792481	2.792481	65	0
Apoa4	2.745927	2.745927	13	0
Fcamr	2.696159	2.196159	0	35
Gaa	2.696159	2.696159	3	9
Clu	2.685779	2.554262	81	129
Abca13	2.660964	2.660964	0	12
Clstn1	2.660964	2.660964	0	11
Cpd	2.660964	2.660964	13	0
Serpinb11	2.584963	2.584963	34	18
Agrn	2.564642	2.564642	0	12
Lipa	2.564642	2.564642	11	0
Hspa5	2.522197	1.568752	238	37
Calu	2.453445	1.453445	12	0
Calu	2.453445	1.453445	0	38
Gm2a	2.453445	2.453445	17	0
Itga2	2.453445	2.453445	9	0
Ces2f	2.321928	2.321928	0	9
Lsr	2.321928	2.321928	0	11
Sdf4	2.321928	2.321928	5	13
Calcb	2.292481	1.792481	0	14
Plxnb1	2.292481	2.292481	12	3
Tgfb2	2.292481	2.292481	6	13
Ctsc	2.160964	2.160964	0	10
Dag1	2.160964	2.160964	8	0
Naga	2.160964	2.160964	5	8
Nenf	2.160964	1.368483	15	0
Sema3d	2.160964	2.160964	7	0
Thbs1	2.160964	2.160964	0	18
Plod3	2.084963	2.084963	4	3
Ccl20	2	2	0	20
Fetub	2	2	7	14
Glycam1	2	2	0	9
Icam1	2	2	0	19
Ppib	2	1.5	59	0
Rnaset2b	2	2	0	16
Tnc	2	2	0	12
Hp	1.953445	1.453445	27	0
Lbp	1.953445	1.953445	1	42
Cp	1.903677	1.611196	0	303
Asah1	1.792481	1.792481	8	0
Dsc2	1.792481	1.792481	0	21
Env1	1.792481	1.792481	11	0
Grn	1.792481	1.792481	0	6
Pam	1.792481	1.792481	0	38
Ppt1	1.792481	1.792481	4	1
Prnp	1.792481	1.292481	15	0
Sema3a	1.792481	1.792481	0	5
UPF0764	1.792481	1.792481	9	0
Tf/Trf	1.77216	1.111196	11	31
Ecm1	1.660964	0.868483	0	68
Pon1	1.660964	1.660964	15	67
Ecm1	1.453445	0.660964	0	3
B2m	1.403677	1.403677	22	0

Lgi2	1.292481	2.292481	0	40
Sulf2	1.292481	1.792481	6	0
Dsg1b	1.216056	2.043731	0	26
Sftpa1	0.868483	2.160964	0	49
Cdh1	0.696159	2.696159	0	27

Table S10. Gene Set Enrichment Analysis of the M cell secretome for markers of cell type using the PanglaoDB Augmented 2021 Database. Related to Figure 3.

term	p value	q value	overlap_genes
Microfold Cells	2.71E-08	3.15E-06	GRN, SPINT1, LAMB3, CCL20, CTSH, CD14, MFGE8, CTSD, ICAM1
Pulmonary Alveolar Type II Cells	6.99E-07	4.05E-05	PIGR, SPINT1, LAMB3, SFTPD, CTSH, CD14, SFTPA1, LTF
Epithelial Cells	2.83E-06	1.09E-04	PIGR, SPINT1, LAMB3, CDH1, CCL20, ITGA2, LAMC2, ICAM1, LTF
Ductal Cells	7.45E-06	2.16E-04	PIGR, MMP7, LAMB3, CDH1, LAMA3, CTSH, LAMC2
Spermatozoa	2.64E-05	6.12E-04	PIGR, CD74, ASAH1, CD14, CP, CLN5

Table S11. Gene Set Enrichment Analysis of the M cell secretome for markers of subcellular compartments using the GO Cellular Component 2025 Database. Related to Figure 3.

term	p value	q value	genes
Collagen-Containing Extracellular Matrix (GO:0062023)	1.69E-14	1.18E-12	LGALS3BP, TGFB2, ECM1, LAMB3, PLOD3, LAMC2, APOA4, L1CAM, CLU, THBS1, ICAM1, CTSL, CHADL, DAG1, CTSH, MFGE8, AGRN, CTSD, CTSC, MATN2
Endoplasmic Reticulum Lumen (GO:0005788)	1.92E-14	1.18E-12	APP, POGLUT1, HSPA5, APLP2, SERPINA10, APOA4, MSLN, CP, THBS1, TF, DAG1, CALU, NUCB1, CALR, PPIB, MFGE8, B2M, CTSC
Lytic Vacuole (GO:0000323)	1.65E-13	6.76E-12	CTSA, APP, CD74, GRN, ASAH1, GAA, SFTPD, CLN5, MAN2B2, CTSL, PPT1, CTSH, MAN2B1, CTSD, LDLR, CTSC
Lysosomal Lumen (GO:0043202)	1.18E-12	3.63E-11	CTSA, CD74, ASAH1, GM2A, CTSL, MAN2B2, GAA, PPT1, MAN2B1, AGRN, CTSD
Intracellular Organelle Lumen (GO:0070013)	1.86E-12	4.58E-11	APP, ASAH1, POGLUT1, SERPINA10, MSLN, THBS1, CTSL, DAG1, CTSH, SDF4, B2M, CTSD, LDLR, CTSC, HSPA5, APLP2, APOA4, CP, TF, CALU, NUCB1, CALR, PPIB, MFGE8, AGRN, LTF
Vacuolar Lumen (GO:0005775)	4.55E-12	9.33E-11	CTSA, CD74, GRN, ASAH1, GAA, GM2A, MAN2B2, CTSL, PPT1, MAN2B1, AGRN, CTSD, CTSC
Lysosome (GO:0005764)	7.39E-12	1.30E-10	CTSA, APP, CD74, GRN, ASAH1, GAA, SFTPD, CP, CLN5, GM2A, MAN2B2, CTSL, PPT1, CTSH, MAN2B1, AGRN, B2M, CTSD, LDLR, CTSC
Secretory Granule Lumen (GO:0034774)	1.42E-11	2.18E-10	CTSA, LGALS3BP, APP, TGFB2, ECM1, GRN, CLU, THBS1, TF, GM2A, CTSH, MAN2B1, B2M, CTSD, CTSC, LTF
Late Endosome (GO:0005770)	3.30E-08	4.52E-07	CD74, TF, GRN, CTSL, SFTPD, SDF4, SFTPA1, B2M, LDLR, SORL1, IGF2R
High-Density Lipoprotein Particle (GO:0034364)	2.93E-06	3.60E-05	APP, PON1, APOA4, CLU

Table S12. Proteins enriched in secretome but absent from scRNA data. Related to Figure 3.

Env1
Agrn
Lgi2
Tgfb2
Gm20547
Matn2
Sema3a
Chadl
Tnc
Sulf2
Hp
Apoa4
Glycam1
Fcamr
Lipa
Tf/Trf
Dsg1b
Mup8
Fetub
Igf2r
UPF0764
Ecm1
Mmp7
Ces2f
Lama3
Man2b2
Ovos
Lamb3
Itga2

Table S13. Proteins enriched in luminal secretome, basolateral secretome, or both. Related to Figure 3.

Basolateral and Luminal	34	Ltf, Fcgbp, Fcamr, Sftpd, Icam1, Tnc, Msln, Tgfb2, Lgals3bp, Poglut1, Lama3, Pigr, Sdf4, Tnfrsf11b, Cd14, H2-Q4, Ctsh, Ecm1, Lamc2, Serpina10, Matn2, Hp, Thbs1, Man2b2, Pam, Vcam1, Il4i1, Gm20547, Psap, Ctsd, Mfge8, Clu, Man2b1, Mmp7
Luminal only	3	Mup8, St6galnac2, Ovos
Basolateral only	71	Serpinb1a, Ppt1, Dag1, Ces2f, Rnaset2b, Agrn, Serpinb11, Plxnb2, Fat1, Hspa5, App, Igf2r, Dsc2, Cd74, H2-K1, Glycam1, Pon1, Tf/Trf, Calu, Asah1, Abca13, Ctsl, Cpd, Env1, Calr, Sorl1, Cp, Dsg2, Ptprk, B2m, Lsr, Plxnb1, Cln5, Ptprz1, Ppib, Lgi2, Nucb2, Cdh1, Clstn1, Sftpa1, Ldlr, Chadl, Plod3, Apoa4, Bpifb1, Sulf2, Sema3a, Naga, Ctsa, Lamb3, Nucb1, Ccl20, Gm2a, Met, Grn, Sema3d, Lbp, H2-D1, L1cam, Lipa, Itga2, Spint1, Calcb, Nenf, Aplp2, Ctsc, Prnp, Fetub, Gaa, UPF0764, Dsg1b
