# Supplementary material for: A sensitive method for profiling cell-type-specific secretomes of rare cells from complex tissues
Source: Cell Rep Methods. 2026 Aug 14;6(10):101552. doi: 10.1016/j.crmeth.2026.101552 (PMC13721748; doi:10.1016/j.crmeth.2026.101552)
Supplement: Supplementary file 2 [file mmc5.pdf]

Cell Reports
Methods

Report

A sensitive method for profiling cell-type-specific secretomes of rare cells from complex tissues

Graphical abstract

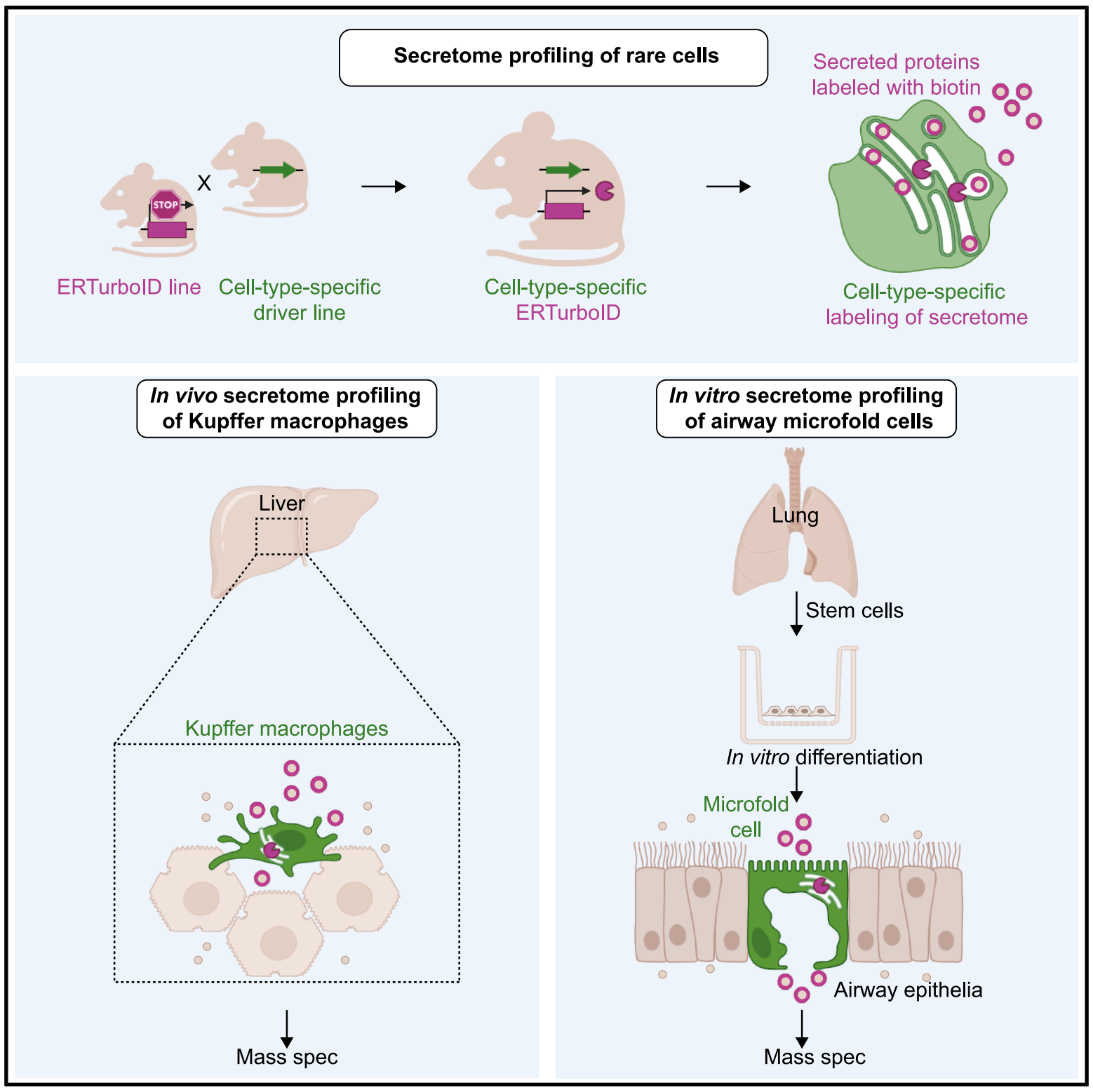

Highlights

- Deposited murine line for cell-type-specific secretome profiling without viral vectors
- Permits sensitive secretome profiling of rare cell types from complex tissues
- Secretomes of rare cells with innate immune functions: Kupffer and microfold cells

Authors

Gergana Shipkovenska, Xuchao Lyu, Manalee Vishnu Surve, ..., Wei Wei, Jonathan Z. Long, Jayaraj Rajagopal

Correspondence

gshipkovenska@mgh.harvard.edu (G.S.), wei.wei2@wisc.edu (W.W.), jzlong@stanford.edu (J.Z.L.), jrajagopal@mgh.harvard.edu (J.R.)

In brief

Shipkovenska et al. present an improved method for profiling cell-type-specific secretomes in complex tissues without the use of viral vectors. The authors perform proof-of-principle validation by profiling the secretomes of two rare cell types with innate immune functions: liver Kupffer cells and airway microfold cells.

Shipkovenska et al., 2026, Cell Reports Methods 6, 101552
October 19, 2026 © 2026 The Authors. Published by Elsevier Inc.
https://doi.org/10.1016/j.crmeth.2026.101552

CellPress

Cell Reports Methods

CellPress
OPEN ACCESS

Report

A sensitive method for profiling cell-type-specific secretomes of rare cells from complex tissues

Gergana Shipkovenska,[1,8,*] Xuchao Lyu,[2,3,8] Manalee Vishnu Surve,[1,8] Joao A. Paulo,[4] Edward Chen,[1] Vanshika Panchal,[1] Brian Lin,[5] Steven P. Gygi,[4] Wei Wei,[6,*] Jonathan Z. Long,[2,3,7,*] and Jayaraj Rajagopal[1,9,*]

[1]Department of Internal Medicine, Division of Pulmonary and Critical Care, Simches 4, Northeast Corridor, Massachusetts General Hospital, Boston, MA 02114, USA
[2]Department of Pathology, Stanford University School of Medicine, Stanford, CA 94305, USA
[3]Sarafan ChEM-H, Stanford University, Stanford, CA 94305, USA
[4]Department of Cell Biology, Harvard Medical School, Boston, MA 02115, USA
[5]Department of Developmental, Molecular and Chemical Biology, School of Medicine, Tufts University, Boston, MA 02111, USA
[6]Department of Biochemistry, University of Wisconsin-Madison, Madison, WI 53706, USA
[7]The Phil & Penny Knight Initiative for Brain Resilience at the Wu Tsai Neurosciences Institute, Stanford University, Stanford, CA 94305, USA
[8]These authors contributed equally
[9]Lead contact
*Correspondence: gshipkovenska@mgh.harvard.edu (G.S.), wei.wei2@wisc.edu (W.W.), jzlong@stanford.edu (J.Z.L.), jrajagopal@mgh.harvard.edu (J.R.)
https://doi.org/10.1016/j.crmeth.2026.101552

MOTIVATION Proximity-ligation-based methods have enabled the study of secretomes from specific cell types. However, these methods deploy viral vectors to deliver biotin ligases to target cells, which can induce innate immune responses and prevent applications in difficult-to-transduce cell types. Here, we sought to engineer a mouse line that expresses a biotin ligase under the control of a *cre-loxP* recombination system. When crossed to a cell-type-specific driver, the biotin ligase is expressed in the endoplasmic reticulum of target cells. As proof of principle, we profile the secretomes of rare cells with innate immune functions from intact liver and cultured airway.

SUMMARY

Very rare cells often have outsized physiologic functions. However, their low abundance presents a technical challenge for studying their function. Here, we generate a murine model, which enables the profiling of secreted proteomes (secretomes) from physiologically relevant low-abundance cells in complex tissues, without the use of viral vectors. We then deploy this model to profile the secretomes of two rare innate immune cell types: Kupffer macrophages from whole liver and the rare airway microfold (M) cells in cultures of differentiated airway epithelia. The method we have developed is characterized by remarkable sensitivity, capturing cell-specific proteins that were not captured in single-cell RNA sequencing (scRNA-seq) atlases. We have made this genetic secretome mouse model available to the community to empower future studies of cell-to-cell communication, secreted protein function, and biomarker discovery for any given cell type *in vitro* or *in vivo*.

INTRODUCTION

The development of methods for proximity-based labeling of proteins[1] has enabled the unbiased identification of the secreted proteomes of target cells of interest, both in cell cultures and in whole organisms.[2–8] However, profiling of rare cells still presents a challenge despite their outsized physiologic importance. Recently developed proximity-based methods for secretome profiling employ promiscuous biotin ligases that are targeted to the endoplasmic reticulum (ER). When exogenous biotin is supplied, these ligases covalently attach biotin moieties to surface lysines in proteins residing within or transiting through the ER. The biotin-modified proteins can then be purified from cell culture media, extracellular fluid, or plasma and subsequently characterized by mass spectrometry.

We and others[2–4] have previously described viral vectors for delivering ER-targeted biotin ligases *in vitro* and *in vivo*. However, viral vectors have several limitations. Target cells often display

© 2026 The Authors. Published by Elsevier Inc.
This is an open access article under the CC BY license (http://creativecommons.org/licenses/by/4.0/).

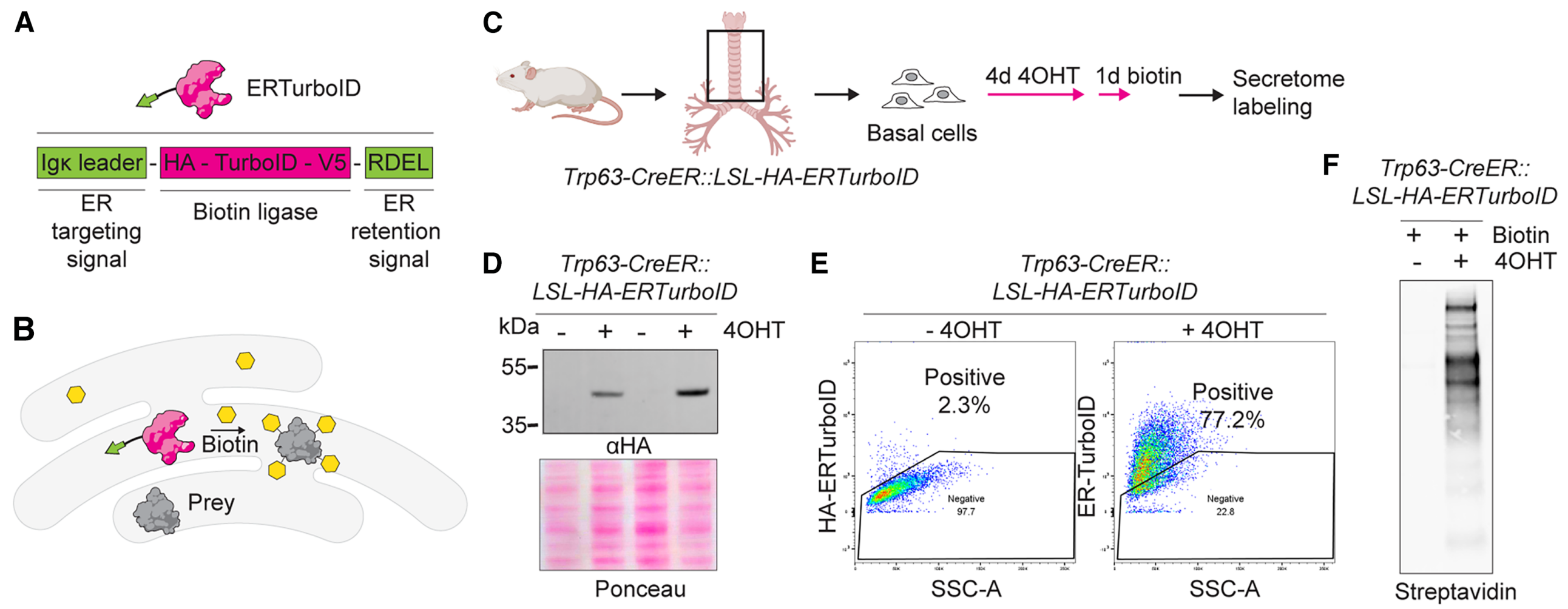

Figure 1. A mouse model for studying cell-type-specific secretomes

(A) Diagram of ERTurboID fusion construct. Biotin ligase TurboID (purple); ER-targeting signals (green).
(B) Diagram of prey (gray) being conjugated to biotin (yellow) inside the lumen of the ER.
(C) Experimental setup for biotin labeling of the secreted proteome of airway basal stem cells.
(D) Immunoblotting for HA-tagged ERTurboID in whole-cell lysates of murine airway basal stem cells.
(E) Flow cytometry analysis of basal stem cell expression of HA-tagged ERTurboID.
(F) Western blot with streptavidin detection of conditioned media from basal stem cells. 4OHT, 4 hydroxy-tamoxifen; LSL, *loxP-STOP-loxP* cassette.
See also Figure S1.

widely varying transduction efficiencies and require the use of particular viral serotypes that differ based on the target cell. Furthermore, vectors must be designed with often ill-defined promoter and enhancer sequences to recapitulate cell-type-specific expression. Finally, the viral vectors themselves introduce artifacts based on the induction of innate immune responses. To circumvent these limitations, we genetically engineered a mouse to conditionally express an ER resident biotin ligase TurboID (ERTurboID) under the control of a *cre-loxP* recombination system. Because labeling sensitivity is a major issue for the detection of secreted proteins from rare cells, we used the biotin ligase with highest activity, TurboID, to build our model.[9] When crossed to a cell-type-specific murine driver line, this enhanced ERTurboID will be expressed in the target population of interest, without the need for exogenous viral delivery.

We validate this reagent by profiling the secreted proteome of a well-studied cell type with a validated cell-specific driver—the liver-resident macrophage (Kupffer cell).[10] We then deployed this technology to map the secretome of a recently described and poorly understood rare cell type in the lung—the airway microfold (M) cell.[11–13] M cells are specialized rare epithelial cells at mucosal surfaces, which uptake luminal antigens and transfer them to the underlying mucosal immune system.[14] Since M cells are highly polarized, we deployed an *in vitro* model of M cell differentiation that preserves the polarity of airway epithelia and allows for the analysis of the basolateral and luminal M cell secretomes separately. We identified a total of 108 M-cell-specific secreted proteins, including known M cell factors, such as TNFRSF11B and CCL20.[11,15] In addition, we identified factors whose expression has not been previously reported in M cells of any organ. Notably, these factors were not captured in previously reported single-cell RNA sequencing (scRNA-seq) M cell profiles.[12] Thus, we establish the utility of our model for *de novo* discovery of physiologically critical pathways in rare cells.

We are making this enhanced *ERTurboID* mouse model available to the broader scientific community to enable high-sensitivity cell-type-specific secretome profiling that can be deployed *in vitro* and in live animals.

RESULTS

Developing a mouse model for cell-type-specific profiling of secreted proteins

We previously generated[2,3] an ERTurboID construct by fusing the biotin ligase TurboID[16] to the ER-targeting Igκ leader sequence and the ER-retention signal KDEL. We have since observed that the RDEL variant of the ER retention signal is more effective at preventing the secretion of TurboID and thereby non-specific biotin labeling. We thus incorporated this variant into our construct (Figure 1A). The ERTurboID ligase localizes to the ER lumen, where it biotinylates resident proteins and proteins transiting *en route* to other secretory compartments, the plasma membrane, or the extracellular space (Figure 1B). We generated a stable mouse line, *LSL-ERTurboID*, carrying the *ERTurboID* cassette inserted into the *H11* "safe harbor" locus and driven by the *pCAG* promoter, followed by a *loxP-STOP-loxP* (LSL) cassette that terminates transcription (Figure S1). In the presence of the tamoxifen-responsive estrogen receptor fusion recombinase (CreER), the two tandem *loxP* sites recombine, resulting in the excision of the transcription termination signal, allowing for expression of the downstream *ERTurboID* cassette (Figure S1).

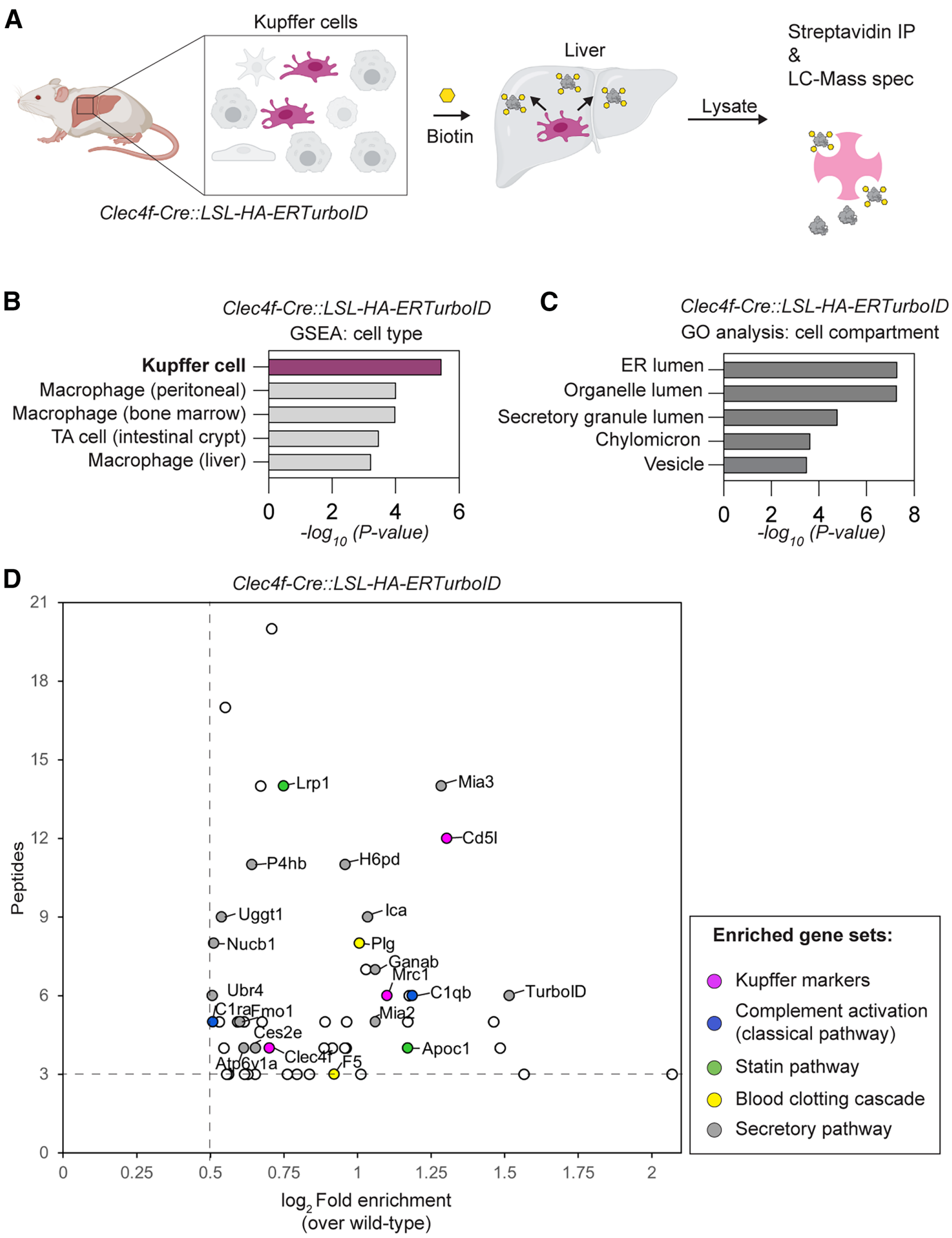

Figure 2. Profiling the secretome of liver Kupffer macrophages in mice *in vivo*

(A) Schematic of experimental design for profiling the secreted proteome of liver Kupffer cells.

(B) Gene set enrichment analysis (GSEA) of the Kupffer cell secretome enriches for terms associated with Kupffer cell and macrophage fates. *p* value was calculated from Fisher exact test.

(C) Gene ontology (GO) term analysis of the Kupffer cell secretome enriches for terms associated with intracellular compartments involved in protein secretion. *p* value was calculated from Fisher's exact test.

(D) Plot of enriched proteins in the Kupffer cell secretome, with color coding according to functional pathways. Legend represents the top five enriched pathways and gene sets.

See also Figure S2 and Tables S1, S3, S5, S7, and S14.

Two alternative secretome mouse models have previously been described: (1) the original ligase BirA* that has lower biotin ligase activity[6] and (2) a variant BirA*G3 with intermediate activity.[8] A head-to-head comparison of the three ligases found that TurboID displayed superior activity in the ER lumen.[9] To first assess this secretome mouse model in an abundant cell type *in vitro*, we crossed the *LSL-ERTurboID* mouse to a *Trp63-CreER* driver, which induces *LSL-ERTurboID* expression specifically in basal stem cells. We dissected tracheas, isolated basal stem cells, and expanded them in culture. We treated basal stem cell cultures with 4-hydroxy-tamoxifen (4OHT) to induce expression of *LSL-ERTurboID* (Figure 1C). Immunoblotting for ERTurboID detected a prominent band at the expected molecular weight of 40.6 kDa in 4OHT-treated basal cell cultures, which was absent in samples from untreated cultures (Figure 1D). Flow cytometry established that 77% of cells expressed ERTurboID following 4OHT treatment as compared to negligible expression without induction (Figure 1E). We then tested the performance of ERTurboID in labeling secreted proteins. To do so, we added biotin to basal stem cell cultures, treated with either 4OHT or vehicle control, collected conditioned medium, and enriched for secreted proteins using streptavidin pulldown. Strong biotinylated protein signal was recovered in 4OHT-treated cultures, confirming that ERTurboID labeling of secreted proteins was highly efficient and specific (Figure 1F).

Using the LSL-ERTurboID mouse model to study secretomes from low-abundance cells *in vivo*

We first profiled the secretomes of liver-resident macrophages, Kupffer cells, *in vivo*. We crossed the *LSL-ERTurboID* line to the Kupffer-specific driver *Clec4f-Cre* (Figure 2A; Table S1).[10] As a comparator, we performed secretome profiling from hepatocytes using viral delivery of *ERTurboID* under the hepatocyte-specific *pTbg* promoter, as previously reported (Figure S2A; Table S2).[2,3,17] Kupffer cells represent less than 5% of the total liver volume.[18] Profiling the Kupffer cell secretome thus presents a challenging use-case scenario for secretome profiling of rare cells in a complex organ environment *in vivo*. Gene set enrichment analysis of the identified secretory proteins from Kupffer cells and hepatocytes showed that the Kupffer cell secretome was enriched for markers of Kupffer and macrophage cell identity, including CLEC4F, MRC1, CD5L, and C1QB (Figure 2B; Table S3), while the hepatocyte secretome was enriched for hepatocyte markers GSTM1, AMBP, ARG1, ASGR1, and CYP7B1 (Figure S2B; Table S4). Furthermore, both secretomes were highly enriched for Gene Ontology (GO) terms associated with the secretory compartment (Figures 2C and S2C; Tables S5 and S6). Pathway

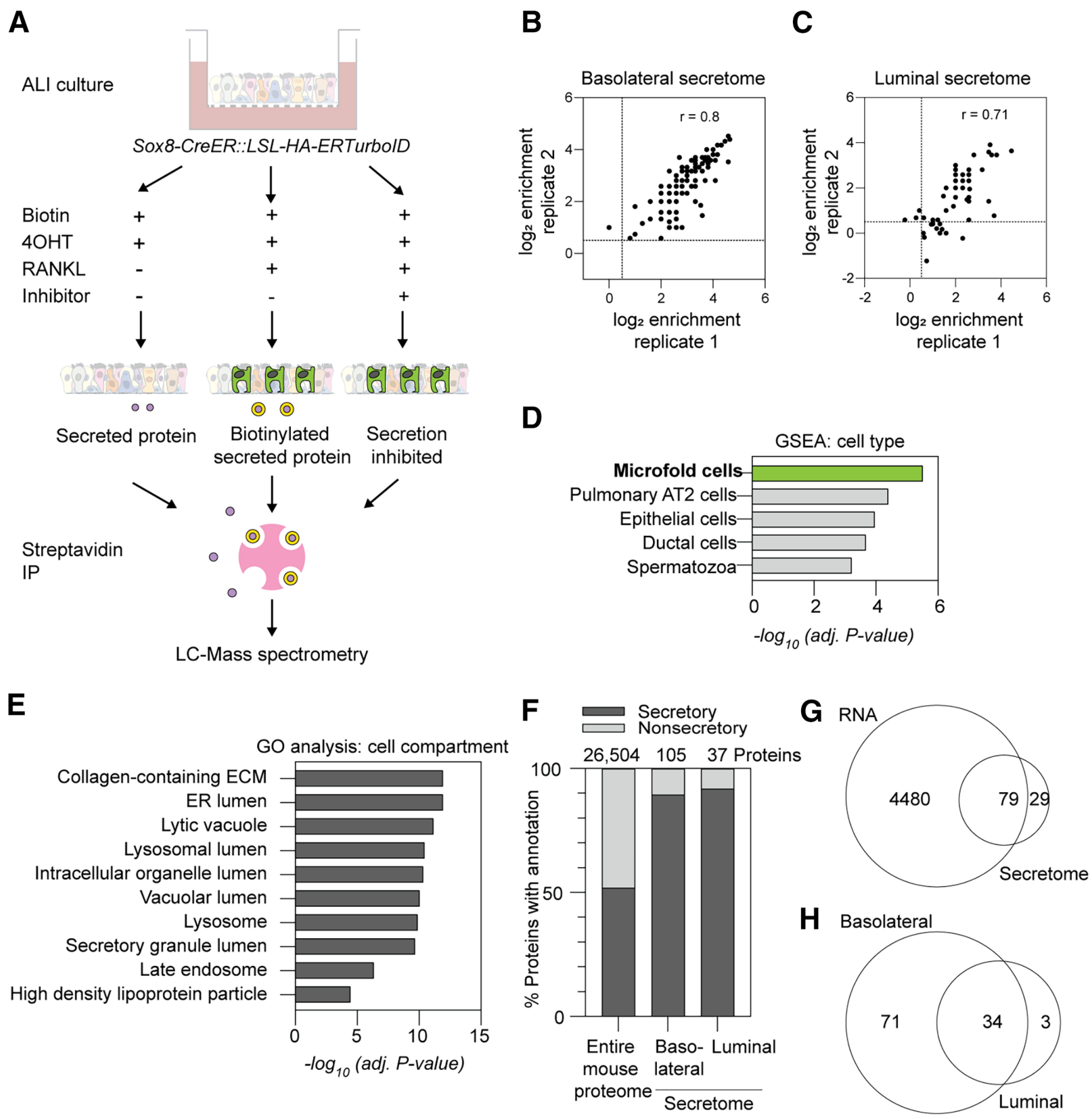

Figure 3. Profiling the secretome of the airway M cell in murine ALI cultures

(A) Schematic of experimental design for profiling the secreted proteome of airway M cells in ALI cultures.

(B) Correlation plot of two biological replicates of the M cell basolateral secretome.

(C) Correlation plot of two biological replicates of the M cell luminal secretome.

(D) Gene set enrichment analysis (GSEA) of the M cell secretome enriches for terms associated with microfold cell fate. p value was calculated from Fisher's exact test and adjusted with Benjamini-Hochberg correction.

(E) Gene ontology (GO) term analysis of the M cell secretome shows enrichment for terms associated with intracellular compartments involved in protein secretion. p value was calculated from Fisher's exact test and adjusted with Benjamini-Hochberg correction.

(F) Annotations from the UniProt database of secreted proteins applied to the M cell secretome. Number of proteins on top of graph. Bars show percentage of proteins with prior secretory annotations in UniProt.

(G) Venn diagram of overlap between genes represented in the transcriptome of airway M cells from single-cell RNA data and proteins represented in the secretome of M cells from this study.

(H) Venn diagram of overlap between the M cell luminal and basolateral secretomes.

See also Figure S3 and Tables S8–S13 and S16.

analysis of the Kupffer cell secretome resulted in enrichment of the "activation of classical complement pathway" (C1QB and C1RA), the "statin pathway" (APOC1 and LRP1), and the "blood clotting cascade" (PLG and F5), represented by proteins with established functions in tissue-resident macrophages and Kupffer cells (Figure 2D; Table S7).[19–23] These results demonstrate that the genetic *LSL-ERTurboID* mouse model has high specificity and sensitivity and can be used as a tool for discovery of physiologically important factors from hard-to-study rare cells in complex tissues.

Defining the secretome of a rare lung cell type, the airway M cell

We next proceeded to profile the secretome of airway M cells, a recently characterized rare lung epithelial cell type.[12,13] M cells are exceedingly rare in the murine lung but can be differentiated using RANKL treatment of air-liquid interface (ALI) cultures where they comprise 5% of all cultured epithelial cells.[12] The transcription factor SOX8 has a well-established role in M cell fate specification[24] and is uniquely expressed by M cells in the airway epithelium.[12] We thus created an M-cell-specific driver *Sox8-CreER* line and crossed it to the *LSL-ERTurboID* secretome line. We expanded basal cells from *Sox8-CreER::LSL-ERTurboID* tracheas and differentiated epithelia in culture (Figure S3A). We then induced M cells by the addition of the cytokine RANKL and further treated the RANKL-induced epithelia with 4OHT to drive the recombination of the *LSL-ERTurboID* allele. We evaluated the specificity and sensitivity of the *Sox8-CreER* driver by co-staining for (1) ERTurboID, (2) SOX8, and (3) a second marker, the cytokine CCL9, which is M cell specific within the airway epithelium[12] (Figures S3B–S3E). The expression of SOX8, CCL9, and ERTurboID in the control cultures where RANKL was omitted was negligible (less than 0.01% of cells stained positive for each marker). By contrast, the expression of SOX8 and CCL9 was strongly induced in RANKL-treated ALI cultures and co-localized in the majority of positive cells as expected. Approximately 28% of SOX8-positive cells and 23% of CCL9-positive cells expressed ERTurboID (Figures S3C and S3D). Expression of ERTurboID in SOX8- or CCL9-negative cells was rare: only 2.8% and 2.4% of all ERTurboID positive cells were SOX8-negative and CCL9-negative, respectively (Figure S3E).

We then proceeded with biotin labeling of the M cell secretome. We treated ALI cultures with RANKL to induce M cell differentiation, followed by 4OHT to induce recombination of *LSL-ERTurboID* (Figure 3A). We then supplemented culture medium with biotin to initiate labeling (Figure 3A). To recover the biotin-labeled secreted proteins, we collected conditioned medium from the bottom compartment of each ALI transwell for the basolateral secretome and washed the epithelial surface of

the top compartment to obtain the luminal secretome. This approach recovers protein products of the conventional secretory pathway,[25] in which secreted proteins transit from the ER through Golgi to be released on the cell surface. We reasoned that the recovery of such proteins would be sensitive to the addition of secretion inhibitors, which disrupt protein trafficking from the ER to the Golgi.[26] Thus, as an additional control for protein secretion, our experimental design included a set of ALI cultures treated with inhibitors of secretion, Brefeldin A and Monensin. Finally, we purified the biotinylated proteins from both normal and secretion-inhibited epithelia using streptavidin pulldowns from basolateral media and luminal washes. We then identified enriched proteins using mass spectrometry (Figure 3A).

We defined the secreted proteome of each sample as the subset of hits, which showed enrichment over both the vehicle control (no RANKL treatment to induce M cell differentiation) and the secretion inhibitor control. The two biological replicates from two independently derived mouse lines were highly correlated (Figures 3B and 3C), with over 98% of hits enriched in both replicates of the basolateral secretome and over 95% of hits enriched in both replicates of the luminal secretome. The few hits that were not shared between replicates were concentrated in the subset of proteins with the lowest enrichment scores. These were excluded from downstream analysis. The M cell proteins were comprised of 37 proteins from the luminal samples and 105 proteins from the basolateral samples (Tables S8 and S9). GO term analysis of the combined set showed strong enrichment for terms associated with M cell fate (Figure 3D; Table S10) and the secretory pathway (Figure 3E; Table S11).

We next asked if the set of proteins recovered by our screen was indeed enriched for proteins known to be secreted. UniProt is a comprehensive database of known proteins with predicted or experimentally validated annotations for all subcellular locations including the secretory compartment. We extracted the UniProt annotations of the subcellular localization of our hits. In the basolateral secretome, 94 out of 105 hits were annotated as secreted, while in the luminal secretome, 34 out of 37 hits were annotated as secreted (Figure 3F). Manual review of the remaining hits using the GeneCards database, which contains annotations of known human genes, revealed that human orthologues of these proteins were validated or predicted to be secreted proteins.

We next asked how well our M cell secretome was represented in our previously published single-cell transcriptomic atlas.[12] Despite the utility of single-cell transcriptomics in studying rare cell populations, sequence dropouts are a well-known caveat. We were thus interested in establishing whether our secretome analysis detects secreted proteins absent in the transcriptomic atlas. We used our published single-cell transcript dataset from mouse trachea, which contained 58 M cells and established an M cell gene expression signature. We used a liberal cutoff for gene expression to capture even those genes with very low expression (see Methods). In total, 29 of the 108 secreted proteins were absent in scRNA-seq profiles (Figure 3G; Table S12). Interestingly, although the basolateral and luminal secretomes showed significant overlap, a sizable subset of proteins was detected solely from either the luminal or basolateral compartment (Figure 3H; Table S13).

Next, we classified the M cell secretome into putative functional categories. As expected, we recovered several components of the secretory pathway (Figure S4A). These proteins transit through the ER *en route* to their respective sub-compartments. We also recovered an extensive list of secreted factors and plasma-membrane-resident proteins (Figures 4A and 4B). The list of secreted proteins included antimicrobial proteins such as opsonins, proteins involved in iron sequestration, and degradation of microbial RNA. Many known components of the extracellular matrix were also identified, as were a number of protease inhibitors and the metalloproteinase MMP7. Additionally, we recovered the cytokines IL4I1 and CCL20 as well as a neuropeptide with known immunomodulatory function, CGRPβ. CCL20 has known functions in the chemotaxis of dendritic cells, B cells, and T cells via its cognate receptor CCR6 and furthermore is required for the formation of lymphoid follicles.[27] In contrast, IL4I1 modulates T effector cells, T regulatory cells, and memory T cell maturation.[28,29] We also recovered TNFRSF11B/osteoprotegerin, a decoy receptor for the M cell differentiation factor RANKL, and validated its expression in M cells by immunofluorescence (Figure S4B).

The list of plasma membrane proteins contains structural factors mediating cell-cell or cell-extracellular matrix adhesion (Figures 4A and 4B). In addition, we recovered a set of cell surface adhesion proteins—VCAM1, ICAM1, and GLYCAM1—which are known to mediate interactions of endothelial cells with immune cells,[30–32] suggesting the possibility that M cells may also interact with immune cells via a similar mechanism. While VCAM1 and ICAM1 were present in our previously published transcriptome atlas, GLYCAM1 was not detected, highlighting the advantage of using multiple complementary profiling approaches. We validate VCAM1 expression in M cells by immunofluorescence (Figure S4C). Furthermore, we recovered cell surface proteins with known functions in antigen presentation (components of the major histocompatibility complexes 1 and 2). Finally, we identified proteins involved in cross-epithelial vesicle trafficking of immunoglobulins: PIGR and FcαμR. PIGR is well known to traffic polymeric immunoglobulin A (IgA) and IgM across epithelia into the luminal space of mucosal organs,[33] while FcαμR is a receptor for IgA and IgM[34] whose presence in airway epithelia was previously unknown. PIGR was detected in the M cell transcriptome, but FcαμR was uniquely identified by secretome profiling.

DISCUSSION

We report the generation of a method for the *de novo* identification of the secreted proteomes of specific cell types of interest *in vivo* and *in vitro*. Our approach is notable for its sensitivity and specificity. Despite the scarcity of rare M cells, which represent only 5% of cells in cultured airway epithelia, we recovered an extensive list of hits, including low-abundance proteins with little or no coverage in transcriptomic data.

Profiling the secretomes of airway M cells highlighted the utility of this method for the *de novo* identification of cell-type-specific secreted factors. We discovered an airway epithelial receptor for secretory IgA (SIgA), FcαμR, expressed specifically in airway M cells. While M cells have long been known to uptake

A

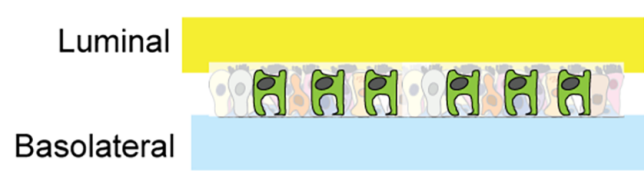

Functional category	Protein hits
Antimicrobial	IL4I1, LBP, CD14, LTF, BPIFB1, SFTPD, RNASET2B, **HP**, SFTPA1, **TF/TRF**, GRN
Cytokines	IL4I1, CCL20
Neuropeptide	CALCB
Proteases	**MMP7**
Protease inhibitors	SERPINB1A, SERPINA10, SPINT1, **FETUB**, SERPINB11, **A2ML1 (OVOS)**
Adherence	VCAM1, ICAM1, **GLYCAM1**,LGALS3BP, MFGE8, CLSTN1, THBS1, DAG1, L1CAM, PLXNB2, PLXNB1, NENF, SEMA3D, **SEMA3A**, FAT1, **ITGA2**, MET, MSLN
Cell-cell junctions	DSG2, DSC2, **DSG1B**, LSR
Extracellular matrix	**MATN2**, **TNC**, **ECM1**, **LAMA3**, **LAMB3**, LAMC2, **CHADL**
MHC I	H2-D1, H2-K1, H2-Q4, B2M
MHC II	CD74
Receptors	PIGR, FCGBP, **FCAMR**, TNFRSF11B, **IGF2R**, PTPRZ1, LDLR, FAT1, CDH1
Secretory pathway	CALR, POGLUT1, HSPA5, CALU, SDF4, PLOD3, NUCB1, NUCB2, CPD, ST6GALNAC2, **MAN2B2**, CTSD, CTSA, CTSH, MAN2B1, CTSL, PSAP, GAA, **LIPA**, GM2A, NAGA, CTSC, PPIB, ASAH1, PPT1, CLN5, **IGF2R**, SORL1, ABCA13
Other	**MUP8**, CLU, **TGFB2**, **APOA4**, **AGRN**, PTPRK, APP, APLP2, PRNP, **GM20547**, **CES2F**, CP, PAM, **UPF0764 PROTEIN C16ORF89 HOMOLOG**, **ENV1**, PON1, **LGI2**, **SULF2**

Proteins highlighted in **bold** were not detected by single cell RNA-seq (Montoro et al, 2018).

B

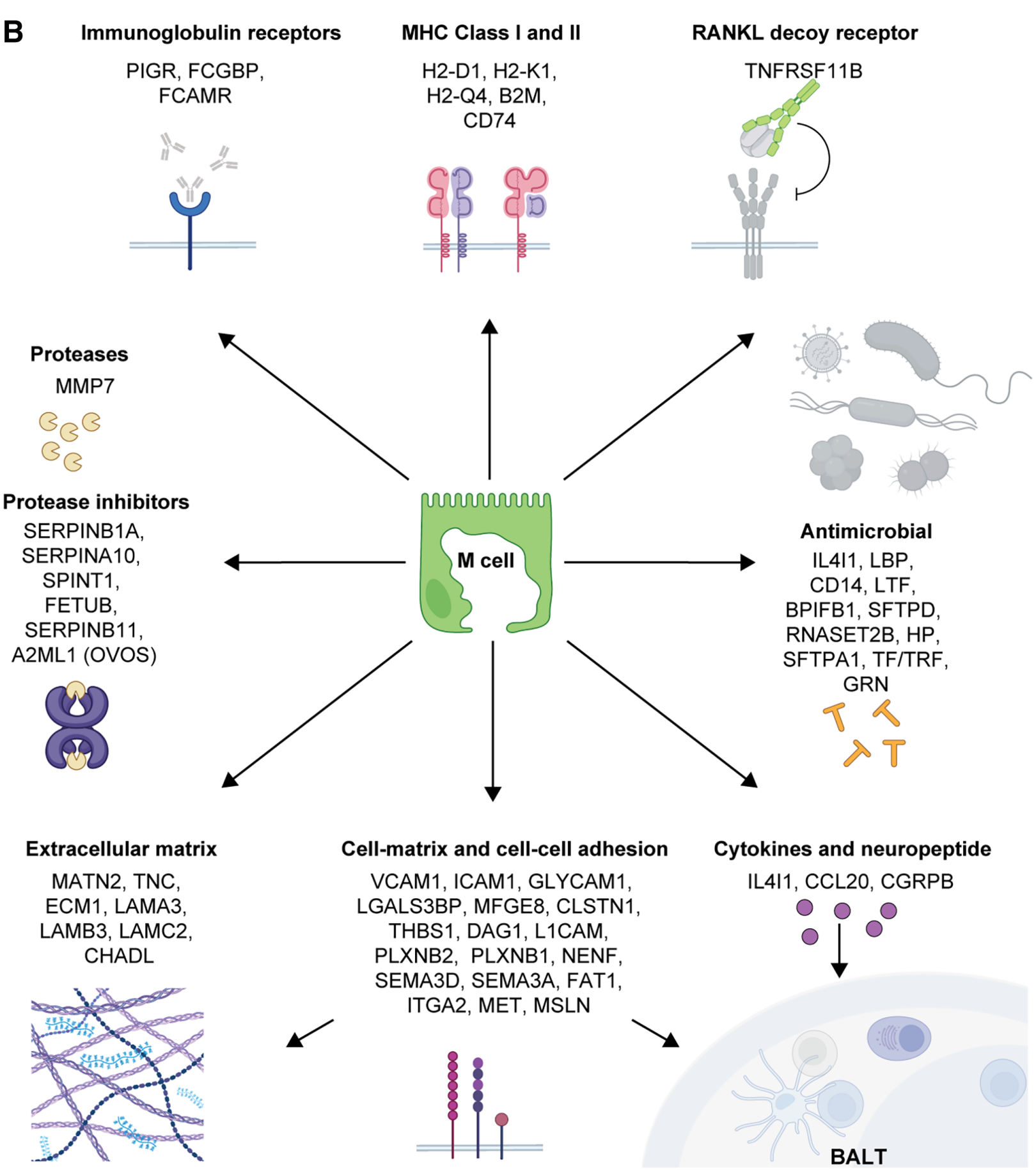

Figure 4. The airway M cell secretome

(A) Table of hits from secretome profiling in airway M cells. Colors represent basolateral (blue), luminal (yellow), or bipolar (no color) secretomes.

(B) Schematic illustrating potential functions of factors secreted by M cells. BALT, bronchus-associated lymphoid tissue.

See also Figure S4.

SIgA-opsonized cargo in the intestine, the receptor was previously unknown. We also identified cell surface adhesion proteins—VCAM1, ICAM1 and GLYCAM1—which may form the basis of an M-cell-immune cell synapse. Finally, we uncovered major histocompatibility complex (MHC) class II proteins in the basolateral secretomes of M cells. M cells have been shown previously to load antigens on MHC II complexes at their basolateral membranes,[35] but our studies further suggest the possibility that M cells may also engage in vesicle-mediated antigen transfer to immune cells. Interestingly, extracellular vesicular MHC class II molecules have previously been described in classical antigen-presenting cells,[36] representing a mechanism for the transfer of processed antigens from the antigen-processing cell to another cell. Given that M cells are exposed to the inhaled environment, we speculate that they function to disseminate processed, MHC-class-II-loaded antigens that are hidden from the immune system.

The deployment of our technology in cultured epithelia also allowed for the characterization of both the luminal and the basolateral secretomes. The basolateral secretome in particular presents a hurdle, as efficient recovery of interstitial fluid from solid tissues is challenging. More sophisticated multichamber systems, such as microfluidic chambers that have been used to separate the neuronal cell body from the axon, could be used for mapping the secretomes of other polarized cell types with subcellular resolution. Furthermore, this technology could be extended to complex culture systems including epithelial-immune cell co-culture platforms.

In the future, the high sensitivity and specificity of this model, coupled with the ability to control the timing of secretome labeling through the administration of tamoxifen and biotin, should allow for sophisticated studies of the dynamics of protein secretion in response to diverse biological perturbations in their appropriate physiologic context.

Limitations of the study

The M cell secretome comprises conventional secreted proteins, cell surface proteins, and proteins in the secretory pathway. The enrichment of all secretome proteins was sensitive to secretion inhibitors, suggesting that cell lysis was not responsible for their presence. The mechanisms of release likely comprise a mixture of conventional protein secretion, ectodomain shedding, and the release of extracellular vesicles. Further studies will be needed to delineate their relative contributions.

RESOURCE AVAILABILITY

Lead contact

Requests for further information and resources should be directed to the lead contact, Jayaraj Rajagopal (jrajagopal@mgh.harvard.edu).

Materials availability

The *LSL-ERTurboID* line is available through Jackson Laboratories (stock no. 041107). The *Sox8-CreER* murine line is available upon request.

Data and code availability

- Raw mass spectrometry data are available via ProteomeXchange with identifier PXD068869. Raw protein quantifications are provided in Tables S14, S15, and S16.
- This paper analyzes existing, publicly available single-cell transcriptomic data, accessible at GEO accession number GSE103354.
- All original code has been deposited at OSF and is publicly available as of the date of publication at https://doi.org/10.17605/OSF.IO/N6CHT.
- Any additional information required to reanalyze the data reported in this paper is available from the lead contact upon request.

ACKNOWLEDGMENTS

The *Trp63-CreER* mouse was a generous gift from Jianming Xu. The *Sox8-CreER* mouse was generated by the Harvard Genome Modification Core Facility. Flow cytometric analysis was performed at the HSCI-CRM Flow Cytometry Core Facility. This work was supported by 2T32HL007633-41 (G.S.), R01HL164563 (J.R.), R01HL157221 (J.R.), UG3CA268117 (J.R.), UH3CA268117 (J.R.), K99HL171884 (M.V.S.), Cystic Fibrosis Foundation Lin19F0 (B.L.), NIDCD R01DC022650 (B.L.), NIH DK130641 (J.Z.L.), OD034455 (J.Z.L.), NIH/NIGMS grant R01 GM132129 (J.A.P.), R35 GM156406 (J.A.P.), Wu Tsai Human Performance Alliance Postdoctoral Fellowship (X.L.), Startup fund from Department of Biochemistry, UW-Madison (W.W.), ADRC at UW-Madison NIH P50AG033514 (W.W.), and DRC at Washington University NIH P30DK020579 (W.W.).

AUTHOR CONTRIBUTIONS

G.S., M.V.S., X.L., W.W., J.Z.L., and J.R. designed the study. B.L. generated the *Sox8-CreER* driver line. J.A.P. performed all proteomic experiments. W.W., G.S., M.V.S., X.L., E.C., and V.P. conducted all other experiments, analyzed the data, interpreted the results, and designed the figures. The manuscript was drafted by G.S. and J.R. J.R., J.Z.L., W.W., S.P.G., J.A.P., and M.V.S. secured the funding. All authors read and approved the final version of the manuscript.

DECLARATION OF INTERESTS

B.L. is a consultant for Rhino Therapeutics and a co-founder of Cellsor Inc. Neither has relevance to this publication. X.L. is currently an employee of Altos Labs, which has no relevance to this publication.

STAR★METHODS

Detailed methods are provided in the online version of this paper and include the following:

- KEY RESOURCES TABLE
- EXPERIMENTAL MODEL AND STUDY PARTICIPANT DETAILS
  - Mouse models
  - Primary cell lines
- METHOD DETAILS
  - Secretome labeling and isolation from basal cells for western blot
  - AAV production
  - Viral transduction
  - *In vivo* biotin labeling
  - Tissue collection
  - Isolation of biotinylated liver proteins
  - Secretome labeling and isolation from microfold cells for proteomics
  - Protein digestion and preparation of mass spectrometry analysis
  - Mass spectrometric data collection
  - Whole-cell lysis and immunoblotting
  - Flow cytometry
  - Immunofluorescence
- QUANTIFICATION AND STATISTICAL ANALYSIS
  - Mass spectrometric data processing
  - Analysis of secretome data from Kupffer cells and hepatocytes
  - Analysis of secretome data from M cells
  - Gene set enrichment analysis
  - Subcellular localization annotations
  - Comparison of secretomes and transcriptomes from M cells

- ○ Immunofluorescence colocalization
- ○ Figures
- ○ Raw proteomic data

SUPPLEMENTAL INFORMATION

Supplemental information can be found online at https://doi.org/10.1016/j.crmeth.2026.101552.

Received: September 29, 2025
Revised: April 20, 2026
Accepted: July 15, 2026
Published: August 14, 2026

REFERENCES

1. Kim, D.I., and Roux, K.J. (2016). Filling the Void: Proximity-Based Labeling of Proteins in Living Cells. Trends Cell Biol. *26*, 804–817. https://doi.org/10.1016/j.tcb.2016.09.004.
2. Wei, W., Riley, N.M., Yang, A.C., Kim, J.T., Terrell, S.M., Li, V.L., Garcia-Contreras, M., Bertozzi, C.R., and Long, J.Z. (2021). Cell type-selective secretome profiling in vivo. Nat. Chem. Biol. *17*, 326–334. https://doi.org/10.1038/s41589-020-00698-y.
3. Wei, W., Riley, N.M., Lyu, X., Shen, X., Guo, J., Raun, S.H., Zhao, M., Moya-Garzon, M.D., Basu, H., Sheng-Hwa Tung, A., et al. (2023). Organism-wide, cell-type-specific secretome mapping of exercise training in mice. Cell Metab. *35*, 1261–1279.e11. https://doi.org/10.1016/j.cmet.2023.04.011.
4. Kim, K.E., Park, I., Kim, J., Kang, M.-G., Choi, W.G., Shin, H., Kim, J.-S., Rhee, H.-W., and Suh, J.M. (2021). Dynamic tracking and identification of tissue-specific secretory proteins in the circulation of live mice. Nat. Commun. *12*, 5204. https://doi.org/10.1038/s41467-021-25546-y.
5. Droujinine, I.A., Meyer, A.S., Wang, D., Udeshi, N.D., Hu, Y., Rocco, D., McMahon, J.A., Yang, R., Guo, J., Mu, L., et al. (2021). Proteomics of protein trafficking by in vivo tissue-specific labeling. Nat. Commun. *12*, 2382. https://doi.org/10.1038/s41467-021-22599-x.
6. Liu, J., Jang, J.Y., Pirooznia, M., Liu, S., and Finkel, T. (2021). The secretome mouse provides a genetic platform to delineate tissue-specific in vivo secretion. Proc National Acad Sci *118*, e2005134118. https://doi.org/10.1073/pnas.2005134118.
7. Qin, W., Cheah, J.S., Xu, C., Messing, J., Freibaum, B.D., Boeynaems, S., Taylor, J.P., Udeshi, N.D., Carr, S.A., and Ting, A.Y. (2023). Dynamic mapping of proteome trafficking within and between living cells by TransitID. Cell *186*, 3307–3324.e30. https://doi.org/10.1016/j.cell.2023.05.044.
8. Yang, R., Meyer, A.S., Droujinine, I.A., Udeshi, N.D., Hu, Y., Guo, J., McMahon, J.A., Carey, D.K., Xu, C., Fang, Q., et al. (2022). A genetic model for in vivo proximity labelling of the mammalian secretome. Open Biol. *12*, 220149. https://doi.org/10.1098/rsob.220149.
9. May, D.G., Scott, K.L., Campos, A.R., and Roux, K.J. (2020). Comparative Application of BioID and TurboID for Protein-Proximity Biotinylation. Cells *9*, 1070. https://doi.org/10.3390/cells9051070.
10. Sakai, M., Troutman, T.D., Seidman, J.S., Ouyang, Z., Spann, N.J., Abe, Y., Ego, K.M., Bruni, C.M., Deng, Z., Schlachetzki, J.C.M., et al. (2019). Liver-Derived Signals Sequentially Reprogram Myeloid Enhancers to Initiate and Maintain Kupffer Cell Identity. Immunity *51*, 655–670.e8. https://doi.org/10.1016/j.immuni.2019.09.002.
11. Kimura, S., Mutoh, M., Hisamoto, M., Saito, H., Takahashi, S., Asakura, T., Ishii, M., Nakamura, Y., Iida, J., Hase, K., and Iwanaga, T. (2019). Airway M Cells Arise in the Lower Airway Due to RANKL Signaling and Reside in the Bronchiolar Epithelium Associated With iBALT in Murine Models of Respiratory Disease. Front. Immunol. *10*, 1323. https://doi.org/10.3389/fimmu.2019.01323.
12. Surve, M.V., Lin, B., Reedy, J.L., Crossen, A.J., Xu, A., Klein, B.S., Vyas, J.M., and Rajagopal, J. (2023). Single-Cell Transcriptomes, Lineage, and Differentiation of Functional Airway Microfold Cells. Am. J. Respir. Cell Mol. Biol. *69*, 698–701. https://doi.org/10.1165/rcmb.2023-0292le.
13. Barr, J., Xu, L., Li, R., Verheyden, J., and Sun, X. (2024). Airway Microfold Cells Emerge in the Post-Influenza Lung. Am. J. Respir. Cell Mol. Biol. *70*, 322–325. https://doi.org/10.1165/rcmb.2023-0351le.
14. Kraehenbuhl, J.-P., and Neutra, M.R. (2000). EPITHELIAL M CELLS: Differentiation and Function. Annu. Rev. Cell Dev. Biol. *16*, 301–332. https://doi.org/10.1146/annurev.cellbio.16.1.301.
15. Kimura, S., Nakamura, Y., Kobayashi, N., Shiroguchi, K., Kawakami, E., Mutoh, M., Takahashi-Iwanaga, H., Yamada, T., Hisamoto, M., Nakamura, M., et al. (2020). Osteoprotegerin-dependent M cell self-regulation balances gut infection and immunity. Nat. Commun. *11*, 234. https://doi.org/10.1038/s41467-019-13883-y.
16. Branon, T.C., Bosch, J.A., Sanchez, A.D., Udeshi, N.D., Svinkina, T., Carr, S.A., Feldman, J.L., Perrimon, N., and Ting, A.Y. (2018). Efficient proximity labeling in living cells and organisms with TurboID. Nat. Biotechnol. *36*, 880–887. https://doi.org/10.1038/nbt.4201.
17. Wei, W., Riley, N.M., Lyu, X., Bertozzi, C.R., and Long, J.Z. (2021). Protocol for cell type-specific labeling, enrichment, and proteomic profiling of plasma proteins in mice. STAR Protoc. *2*, 101014. https://doi.org/10.1016/j.xpro.2021.101014.
18. Mendoza, R., Banerjee, I., Reghupaty, S.C., Yetirajam, R., Manna, D., and Sarkar, D. (2022). Non-Alcoholic Steatohepatitis, Methods and Protocols. Methods Mol. Biol. *2455*, 73–84. https://doi.org/10.1007/978-1-0716-2128-8_7.
19. Lu, J., and Kishore, U. (2017). C1 Complex: An Adaptable Proteolytic Module for Complement and Non-Complement Functions. Front. Immunol. *8*, 592. https://doi.org/10.3389/fimmu.2017.00592.
20. Xian, X., Ding, Y., Dieckmann, M., Zhou, L., Plattner, F., Liu, M., Parks, J.S., Hammer, R.E., Boucher, P., Tsai, S., and Herz, J. (2017). LRP1 integrates murine macrophage cholesterol homeostasis and inflammatory responses in atherosclerosis. eLife *6*, e29292. https://doi.org/10.7554/elife.29292.
21. Westerterp, M., Van Eck, M., de Haan, W., Offerman, E.H., Van Berkel, T.J.C., Havekes, L.M., and Rensen, P.C.N. (2007). Apolipoprotein CI aggravates atherosclerosis development in ApoE-knockout mice despite mediating cholesterol efflux from macrophages. Atherosclerosis *195*, e9–e16. https://doi.org/10.1016/j.atherosclerosis.2007.01.015.
22. Mak, P.A., Laffitte, B.A., Desrumaux, C., Joseph, S.B., Curtiss, L.K., Mangelsdorf, D.J., Tontonoz, P., and Edwards, P.A. (2002). Regulated Expression of the Apolipoprotein E/C-I/C-IV/C-II Gene Cluster in Murine and Human Macrophages A CRITICAL ROLE FOR NUCLEAR LIVER X RECEPTORS α AND β. J. Biol. Chem. *277*, 31900–31908. https://doi.org/10.1074/jbc.m202993200.
23. Zhang, N., Czepielewski, R.S., Jarjour, N.N., Erlich, E.C., Esaulova, E., Saunders, B.T., Grover, S.P., Cleuren, A.C., Broze, G.J., Edelson, B.T., et al. (2019). Expression of factor V by resident macrophages boosts host defense in the peritoneal cavity. J. Exp. Med. *216*, 1291–1300. https://doi.org/10.1084/jem.20182024.
24. Kimura, S., Kobayashi, N., Nakamura, Y., Kanaya, T., Takahashi, D., Fujiki, R., Mutoh, M., Obata, Y., Iwanaga, T., Nakagawa, T., et al. (2019). Sox8 is essential for M cell maturation to accelerate IgA response at the early stage after weaning in mice. J. Exp. Med. *216*, 831–846. https://doi.org/10.1084/jem.20181604.
25. Rabouille, C. (2017). Pathways of Unconventional Protein Secretion. Trends Cell Biol. *27*, 230–240. https://doi.org/10.1016/j.tcb.2016.11.007.
26. Dinter, A., and Berger, E.G. (1998). Golgi-disturbing agents. Histochem. Cell Biol. *109*, 571–590. https://doi.org/10.1007/s004180050256.
27. WILLIAMS, I.R. (2006). CCR6 and CCL20. Ann. N. York Acad. Sci. *1072*, 52–61. https://doi.org/10.1196/annals.1326.036.
28. Romagnani, S. (2016). IL4I1: Key immunoregulator at a crossroads of divergent T-cell functions. Eur. J. Immunol. *46*, 2302–2305. https://doi.org/10.1002/eji.201646617.

29. Puiffe, M.-L., Dupont, A., Sako, N., Gatineau, J., Cohen, J.L., Mestivier, D., Lebon, A., Prévost-Blondel, A., Castellano, F., and Molinier-Frenkel, V. (2020). IL4I1 Accelerates the Expansion of Effector CD8+ T Cells at the Expense of Memory Precursors by Increasing the Threshold of T-Cell Activation. Front. Immunol. *11*, 600012. https://doi.org/10.3389/fimmu.2020.600012.

30. Lasky, L.A., Singer, M.S., Dowbenko, D., Imai, Y., Henzel, W.J., Grimley, C., Fennie, C., Gillett, N., Watson, S.R., and Rosen, S.D. (1992). An endothelial ligand for L-Selectin is a novel mucin-like molecule. Cell *69*, 927–938. https://doi.org/10.1016/0092-8674(92)90612-g.

31. Elices, M.J., Osborn, L., Takada, Y., Crouse, C., Luhowskyj, S., Hemler, M.E., and Lobb, R.R. (1990). VCAM-1 on activated endothelium interacts with the leukocyte integrin VLA-4 at a site distinct from the VLA-4/Fibronectin binding site. Cell *60*, 577–584. https://doi.org/10.1016/0092-8674(90)90661-w.

32. Marlin, S.D., and Springer, T.A. (1987). Purified intercellular adhesion molecule-1 (ICAM-1) is a ligand for lymphocyte function-associated antigen 1 (LFA-1). Cell *51*, 813–819. https://doi.org/10.1016/0092-8674(87)90104-8.

33. Kaetzel, C.S. (2005). The polymeric immunoglobulin receptor: bridging innate and adaptive immune responses at mucosal surfaces. Immunol. Rev. *206*, 83–99. https://doi.org/10.1111/j.0105-2896.2005.00278.x.

34. Sakamoto, N., Shibuya, K., Shimizu, Y., Yotsumoto, K., Miyabayashi, T., Sakano, S., Tsuji, T., Nakayama, E., Nakauchi, H., and Shibuya, A. (2001). A novel Fc receptor for IgA and IgM is expressed on both hematopoietic and non-hematopoietic tissues. Eur. J. Immunol. *31*, 1310–1316. https://doi.org/10.1002/1521-4141(200105)31:5<1310::aid-immu1310>3.0.co;2-n.

35. Wang, D., Lim, S., Wetering, W.J. van de, Lopez-Iglesias, C., Okura, Y., Teranishi-Ikawa, Y., Mizoroki, A., Spoelstra, W.K., Dayton, T., Son, G.J.F., et al. (2025). Human gut M cells resemble dendritic cells and present gluten antigen. Nature *650*, 251–260. https://doi.org/10.1038/s41586-025-09829-8.

36. Buzas, E.I. (2023). The roles of extracellular vesicles in the immune system. Nat. Rev. Immunol. *23*, 236–250. https://doi.org/10.1038/s41577-022-00763-8.

37. Lee, D.-K., Liu, Y., Liao, L., Wang, F., and Xu, J. (2014). The Prostate Basal Cell (BC) Heterogeneity and the p63-Positive BC Differentiation Spectrum in Mice. Int. J. Biol. Sci. *10*, 1007–1017. https://doi.org/10.7150/ijbs.9997.

38. Xie, Z., Bailey, A., Kuleshov, M.V., Clarke, D.J.B., Evangelista, J.E., Jenkins, S.L., Lachmann, A., Wojciechowicz, M.L., Kropiwnicki, E., Jagodnik, K.M., et al. (2021). Gene Set Knowledge Discovery with Enrichr. Curr. Protoc. *1*, e90. https://doi.org/10.1002/cpz1.90.

39. Hao, Y., Hao, S., Andersen-Nissen, E., Mauck, W.M., Zheng, S., Butler, A., Lee, M.J., Wilk, A.J., Darby, C., Zager, M., et al. (2021). Integrated analysis of multimodal single-cell data. Cell *184*, 3573–3587.e29. https://doi.org/10.1016/j.cell.2021.04.048.

40. Schneider, C.A., Rasband, W.S., and Eliceiri, K.W. (2012). NIH Image to ImageJ: 25 years of image analysis. Nat. Methods *9*, 671–675. https://doi.org/10.1038/nmeth.2089.

41. Lin, B., Shah, V.S., Chernoff, C., Sun, J., Shipkovenska, G.G., Vinarsky, V., Waghray, A., Xu, J., Leduc, A.D., Hintschich, C.A., et al. (2024). Airway hillocks are injury-resistant reservoirs of unique plastic stem cells. Nature *629*, 869–877. https://doi.org/10.1038/s41586-024-07377-1.

42. Elias, J.E., and Gygi, S.P. (2007). Target-decoy search strategy for increased confidence in large-scale protein identifications by mass spectrometry. Nat. Methods *4*, 207–214. https://doi.org/10.1038/nmeth1019.

43. Elias, J.E., and Gygi, S.P. (2010). Target-decoy search strategy for mass spectrometry-based proteomics. Methods Mol. Biol. *604*, 55–71. https://doi.org/10.1007/978-1-60761-444-9_5.

STAR★METHODS

KEY RESOURCES TABLE

REAGENT or RESOURCE	SOURCE	IDENTIFIER
Antibodies		
anti-hemagglutinin (HA) antibody, cl. 3F10	Roche	11867423001; RRID: AB_390918
α-SOX8	Santa Cruz	sc-374445; RRID: AB_10988426
α-CCL9	R and D Systems Inc	AF463; RRID: AB_2071817
α-VCAM1, cl. 429/MVCAM.A	BDBiosciences	553330; RRID: AB_394786
α-TNFRSF11B	Biotechne	AF459; RRID: AB_355370
Bacterial and virus strains		
AAV8-p*Tbg-ERTurboID*	Penn Vector Core	Batch #: V7069S
Chemicals, peptides, and recombinant proteins		
Papain	Worthington-Biochem	Cat# LS001220
DNase I	Roche	Cat# 10104159001
SAGM	Lonza	CC-3119
Rocki Y-27632	Selleck Chemicals	S1049
CHIR 99021	Tocris	4423
A8301	Tocris	2939
Primocin	INVIVOGEN	ant-pm-2
Trypsin-LE	Gibco	12605010
Bambanker media	Bulldog-Bio	BB05
6.5mm 0.4 μm polyester membranes Transwell inserts	Corning	3470
PneumaCult ALI medium	StemCell Technologies	05001
(Z)-4-hydroxy Tamoxifen	Cayman Chemical	14854
D-(+)-Biotin ≥97.5%, Cell Culture Grade	VWR	76177–768
Dynabeads MyOne Streptavidin T1 magnetic beads	Thermo Fisher	65602
IRDye® 680RD Streptavidin	Licor	926–68079
Biotin	Sigma	B4501-10G
DMSO	Sigma	D2650-100ML
HALT protease inhibitor	Thermo Scientific	87786
Mouse RANKL (Peprotech)	Thermo Fisher Scientific	315-11C-10UG
eBioscience™ Protein Transport Inhibitor Cocktail	Thermo Fischer Scientific	00-4980-93
Pierce™ Bovine Serum Albumin, Biotinylated	Thermo Fischer Scientific	29130
Critical commercial assays		
EndoFree Qiagen Maxiprep kit	Qiagen	12362
Deposited data		
Raw proteomic data	ProteomeXchange	ProteomeXchange: PXD068869
Experimental models: Cell lines		
Trp63-CreER::LSL-ERTurboID airway basal cell line F1 (female)	This study	N/A
Trp63-CreER::LSL-ERTurboID airway basal cell line M1 (male)	This study	N/A
Sox8-CreER::LSL-ERTurboID airway basal cell line F1 (female)	This study	N/A

(*Continued on next page*)

CellPress
OPEN ACCESS

Continued

REAGENT or RESOURCE	SOURCE	IDENTIFIER
Sox8-CreER::LSL-ERTurboID airway basal cell line M1 (male)	This study	N/A
Experimental models: Organisms/strains		
C57BL/6J-*Igs2tm1(CAG-TurboID)Long*/J	This study; available through Jackson Laboratories	Jax:041107
C57BL/6J	Jackson Laboratories	Jax:000664
Sox8-CreER	This study	N/A
Trp63-CreER	Lee et al.[37]	N/A
Clec4f-Cre	Sakai et al.[9]	Jax: 033296
Oligonucleotides		
ACTCTACTGGAGGAGGACAAACTGGTCAC	Integrated DNA Technologies	H11_WT_FWD
TTGTTCCCTTTCTGCTTCATCTTGCTGA	Integrated DNA Technologies	H11_WT_REV
CTCCACACAGGCATAGAGTGTC	Integrated DNA Technologies	ERTurboID_FWD
GCACGGTGAACACGTCCAC	Integrated DNA Technologies	ERTurboID_REV
TTCCATTCATCCCGTCGGC	Integrated DNA Technologies	P388_Sox8C_GT_WTF
TTTAGCTGGCCCAAATGTTGC	Integrated DNA Technologies	P389_Sox8C_GT_MutR
ATCCAAACCGGAGAGCAACG	Integrated DNA Technologies	P390_Sox8C_GT_WTR
Recombinant DNA		
pAAV-pTBG-ERTurboID	Wei et al.[2]	N/A
Software and algorithms		
Mouse UniProt database (peptide search)	UniProt	Version: January 2023
Mouse UniProtKB database (subcellular localization)	UniProtKB	Version: November 2023
Gene Set Enrichment Analysis	Enrichr[38]	Ref. databases listed below
CellMarker Database	Enrichr	Version: 2024, mouse
GO Cellular Component 2025 Database	Enrichr	Version: 2025
WikiPathways 2024 Mouse Database	Enrichr	Version: 2024
PanglaoDB Augmented Database	Enrichr	Version: 2021
Original R code	OSF	OSF: https://doi.org/10.17605/OSF.IO/N6CHT
MassPike (Software for quantitative proteomics)	Licensed by Harvard Medical School	N/A
Seurat	Hao et al.[39]	Version: 4
Fiji	Schneider et al.[40]	2.16.0/1.54p
FlowJo	Becton, Dickinson and Company	Version: 10.9
R	R Project	Version: 4.3.2
GraphPad Prism	GraphPad Software	Version: 10.5.0
Other		
10kDa filter tube	Millipore	UFC801024
Benchmark BeadBlaster Homogenizer	Benchmark Scientific	D2400
3 kDa filter tubes	Millipore	UFC900324
Exploris 480 mass spectrometer	Thermo Fisher Scientific	N/A
Proxeon 1200 Liquid Chromatograph	Thermo Fisher Scientific	N/A
Accucore C18 resin (2.6 μm, 150 Å)	Thermo Fisher Scientific	N/A
BD FACSAria II system	BD Biosciences	N/A

EXPERIMENTAL MODEL AND STUDY PARTICIPANT DETAILS

Mouse models

The LSL-*ERTurboID* model was generated using TARGATT knock-in technology by Applied Stem Cell. The *ERTurboID* cassette was inserted at the *H11* safe harbor locus on a C57BL/6J background (Jackson Laboratories, stock no. 000664). Genotyping primers are listed below.

H11_WT_FWD	ACTCTACTGGAGGAGGACAAACTGGTCAC
H11_WT_REV	TTGTTCCCTTTCTGCTTCATCTTGCTGA
ERTurboID_FWD	CTCCACACAGGCATAGAGTGTC
ERTurboID_REV	GCACGGTGAACACGTCCAC

The *Sox8-CreER* knock-in mouse was generated using CRISPR-Cas9 microinjection. The targeting construct was designed to knock-in a *GSG-P2A* linked *CreER* sequence immediately downstream of the *Sox8* coding sequence, with a single silent mutation to deactivate the targeting sgRNA. This sequence was flanked with symmetric 300 bp arms of homology and synthesized as an ssODN at Genscript. A single chemically modified guide RNA: ACCACCCTGACCCGACCCTGAGG was purchased from Synthego. Wildtype Cas9 was purchased from IDT. These three components were microinjected as a core service by the Harvard Genome Modification Core Facility into C57bl6 oocytes. Of 26 founder animals, nine were knocked into the correct locus, and four produced proper knock-in bands by long-range PCR and sequencing. These founders were backcrossed for 2 generations before use and genotyped with the following primers.

P388_Sox8C_GT_WTF	TTCCATTCATCCCGTCGGC	380 bp wt
P389_Sox8C_GT_MutR	TTTAGCTGGCCCAAATGTTGC	470 bp Mut
P390_Sox8C_GT_WTR	ATCCAAACCGGAGAGCAACG	–

The *Trp63-CreER* model has been previously described.[37] C57BL/6J male and female mice (stock no. 000664), and male homozygous *Clec4f-Cre* mice (Jackson Laboratory, stock no. 033296) were purchased from The Jackson Laboratory. Mice were housed in an Association for Assessment and Accreditation of Laboratory Animal Care-accredited animal facility at the Massachusetts General Hospital and Administrative Panel on Laboratory Animal Care-accredited facility at Stanford University. All procedures were approved by the MGH and Stanford Institutional Animal Care and Use Committees (IACUC). Standard housing conditions were used throughout, unless otherwise noted (temperature of 68–79°F, humidity of 30–70%, a 12-h light–dark cycle and regular rodent diet).

For secretome profiling from whole liver, 8–12-week-old male mice with the following genotypes were used: *Clec4f-Cre LSL-ERTurboID* and C57BL/6J. For *in vitro* secretome profiling from airway basal stem cells and microfold cells, primary cell lines were established from the tracheas of 8–12-week-old male and female mice with the following genotypes: *Trp63-CreER LSL-ERTurboID* and *Sox8-Cre LSL-ERTurboID.*

Primary cell lines

Basal stem cells from tracheas of *Trp63-CreER::LSL-ERTurboID* and *Sox8-CreER::LSL-ERTurboID* mice were isolated and expanded *in vitro*, as described previously.[41] Briefly, tracheas were dissected and dissociated to single cells using 10U papain (Worthington-Biochem, cat# LS001220) and 100 μg DNase I (Roche, Cat# 10104159001) in dissociation buffer (0.067 mM beta-mercaptoethanol, 1.1 mM EDTA, 5.5 mM Cysteine-HCl, in EBSS with Mg and Ca). The single cell suspension was plated in tissue culture-grade plates, coated with 804G conditioned medium and submerged in SAGM-3I medium (SAGM (Lonza CC-3119), supplemented with 5 μM Rocki Y-27632 (Selleck Chemicals S1049), 1 μM CHIR 99021 (Tocris 4423), 1 μM A8301 (Tocris 2939) and 50mg/mL Primocin (INVIVOGEN, ant-pm-2)). Basal cells were initially expanded to 50% confluence with alternate day media changes, dissociated with Trypsin-LE (Gibco, 12605010) and passaged onto uncoated plates in SAGM-3I. Upon reaching 75% confluence, cells were dissociated with Trypsin-LE and frozen down in Bambanker media (Bulldog-Bio, BB05) for long-term storage. All subsequent cell culture was performed on uncoated plates with alternate day media changes and cells were passaged in 1:8 ratio once reaching 50–75% confluence. All primary cell lines have been tested for Mycobacterial contamination. *In vitro* grown murine airway epithelia from *Sox8-CreER::LSL-ERTurboID* basal stem cells were prepared and differentiated for 14 days on 0.4 μm PET membrane transwells, as described in detail previously.[41] Briefly, *Sox8-CreER::LSL-ERTurboID* basal stem cells were isolated and expanded as described above. Basal cells were then dissociated with Trypsin-LE, 200,000 cells were plated on 6.5mm 0.4 μm polyester

membranes Transwell inserts (Corning, 3470), coated with 804G conditioned medium, and SAGM-3I medium was added to the top and bottom compartments. The submerged Transwell cultured were incubated for 1 day to allow for cell attachment and recovery. Subsequently, medium from the top compartment was removed to establish an air-liquid interface and medium from the bottom compartment was replaced with PneumaCult ALI medium (StemCell Technologies, 05001), supplemented with 50mg/mL Primocin. The air-liquid interface cultures were incubated for 14 days with alternate day media changes to allow for full differentiation and ciliogenesis.

METHOD DETAILS

Secretome labeling and isolation from basal cells for western blot

Trp63-CreER::LSL-ERTurboID basal cells were plated on 10cm plates, allowed to attach for 1 day and treated with either 25nM 4OHT (Cayman Chemical, 14854) or vehicle control for 4 days with daily media changes to induce *LSL-ERTurboID* recombination. On day 5, cultures had reached 60% confluence (~4 million cells). They were washed three times with DPBS to remove old media and incubated with fresh medium, supplemented with 500 μM biotin (VWR, 76177-768) for 24h. Conditioned medium was collected from each plate, centrifuged through a 10kDa filter tube (Millipore UFC801024) at 3220g for 10min to remove small solutes and concentrate proteins and then washed 8 times in the filter tube with 10 times excess volume of PBS per wash to remove any residual biotin, which may interfere with downstream processing. After the last wash, the concentrated sample was diluted in IP buffer (50 mM Tris-HCl, 150 mM NaCl, 0.1% SDS, 0.5% sodium deoxycholate, 1% NP40, 1 mM EDTA, 1× HALT protease inhibitor (Thermo Scientific, 87786)) and incubated with 100μL Dynabeads MyOne Streptavidin T1 magnetic beads (ThermoFisher 65602). Beads were washed twice with IP buffer, once with 1 M KCl, once with 0.1 M Na2CO3, once with 2 M urea in 10 mM Tris-HCl (pH 8.0), and twice with IP buffer. Proteins were eluted from the beads by boiling for 10min in 2× NuPAGE LDS Sample Buffer (Invitrogen NP0007) supplemented with 20 mM DTT and 2 mM biotin. The eluted proteins were analyzed by immunoblotting on a Licor system, using fluorescently conjugated streptavidin for detection (IRDye 680RD Streptavidin, Licor 926–68079).

AAV production

pAAV-TBG-TurboID plasmids were amplified, purified using an EndoFree Qiagen Maxiprep kit (Qiagen, 12362), and sequence-verified. AAVs were packaged by the Penn Vector Core.

Viral transduction

Male mice aged 8–12 weeks were transduced by tail vein injection using a 29-gauge syringe (Thermo Fisher, 14-841-31). Each mouse received 5 × 10 ˆ11 genome copies (GC) of virus diluted in sterile saline to a final volume of 100 μL. One week after viral transduction, mice were used for secretome profiling as outlined below.

In vivo biotin labeling

Three days before tissue collection, mice were subjected to *in vivo* biotin labeling by receiving biotin-supplemented drinking water containing 0.5 mg/mL biotin (Sigma, B4501-10G) and 3% DMSO (Sigma, D2650-100ML) for 3 days.

Tissue collection

Liver tissues were dissected, weighed, transferred into Eppendorf tubes, snap-frozen on dry ice, and stored at −80°C until further analysis.

Isolation of biotinylated liver proteins

To isolate biotinylated liver proteins, liver samples were homogenized in 1 mL ice-cold PBS buffer containing 1× HALT protease inhibitor (Thermo Scientific, 87786) using a Benchmark BeadBlaster Homogenizer at 4°C. Homogenates were centrifuged at 13,000 rpm for 10 min at 4°C to remove insoluble material.

Dynabeads MyOne Streptavidin T1 magnetic beads (Thermo Fisher, 65602; 200 μL per sample) were washed twice with 1 mL washing buffer (50 mM Tris-HCl, 150 mM NaCl, 0.1% SDS, 0.5% sodium deoxycholate, 1% NP-40, 1 mM EDTA, 1× HALT protease inhibitor, 5 mM trolox, 10 mM sodium azide and 10 mM sodium ascorbate) and resuspended in 100 μL washing buffer. The beads were then incubated with liver lysates overnight at 4°C with rotation to capture biotinylated proteins.

Following incubation, beads were washed twice with washing buffer, followed by one wash each with 1 M KCl, 0.1 M Na2CO3, and 2 M urea in 10 mM Tris-HCl (pH 8.0), and then washed twice more with washing buffer. Tubes were vortexed briefly (~3 s) between washing steps. After the final wash, bead-bound proteins were submitted to the Thermo Fisher Center for Multiplexed Proteomics at Harvard Medical School for proteomic analysis.

Secretome labeling and isolation from microfold cells for proteomics

Secretome profiling was performed in two biological replicates in cell lines derived from two *Sox8-CreER::LSL-ERTurboID* mice. For each cell line, 24 6.5mm fully differentiated ALI transwells were grown in PneumaCult ALI complete medium supplemented with either 200ng/mL murine RANKL (Peprotech, ThermoFisher Scientific: 315-11C-10UG) (16 transwells) or vehicle control (8 transwells) for

7 days with daily media changes. During the last 4 days of treatment, media was also supplemented with 1 μM 4OHT to induce *LSL-ERTurboID* recombination in *Sox8-CreER* expressing cells. The cultures were then used for secretome labeling as follows. Transwells were transferred to fresh plates filled with Dulbecco's PBS (DPBS) to wash the bottom surface of each well from any secreted proteins prior to biotin labeling. The top surface of each transwell was washed with 150 μl DPBS for 15min. The DPBS washes were then removed and the transwells were transferred to a fresh plate containing PneumaCult ALI complete medium. To 8 transwells of RANKL treated ALI cultures, a cocktail of inhibitors of the secretory pathway (eBioscience Protein Transport Inhibitor Cocktail, ThermoFischer Scientific, 00-4980-93) was added to the medium for 1h, after which the medium was also supplemented with 500 μM biotin. For the remaining 8 transwells of RANKL treated ALI cultures and for the vehicle treated ALI cultures, 500 μM biotin was added directly to the medium without treatment with secretion inhibitor. All cultures were then incubated with biotin for 16h.

Secretome collection was performed as follows. For luminal secretomes, the top chamber of each Transwell was filled with 50 μl DPBS and incubated for 15min with repeat pipetting to solubilize any secreted proteins. Luminal washes were then pooled across 8 transwells per sample, for a total of 6 luminal samples: two biological replicates each of vehicle control, RANKL treated, and RANKL treated with secretion inhibitor. The 6 luminal samples were centrifuged at 1000g for 5 min to remove any intact cells which might have been dislodged from the surface of the epithelium during the washes. For basolateral secretomes, conditioned medium from the bottom chamber of each transwell was collected and pooled across 8 transwells per sample, for a total of 6 basolateral samples: two biological replicates each of vehicle control, RANKL treated, and RANKL treated with secretion inhibitor.

Secretome purification was performed in parallel on the 6 basolateral and 6 luminal samples as follows. Prior to purification, 10ng of biotinylated BSA (Pierce Bovine Serum Albumin, Biotinylated Thermo Fischer Scientific, 29130) was added to each basolateral and luminal sample as an internal spike-in control. Samples were centrifuged through 3 kDa filter tubes (Millipore UFC900324) at 3220xg for 40min to remove small solutes and concentrate proteins. Samples were then washed 8 times in the filter tubes to remove any residual biotin, which could interfere with downstream processing. After the last wash, the concentrated samples were diluted in IP buffer (50 mM Tris-HCl, 150 mM NaCl, 0.1% SDS, 0.5% sodium deoxycholate, 1% NP40, 1 mM EDTA, 1× HALT protease inhibitor) and incubated with 100 μl Dynabeads MyOne Streptavidin T1 magnetic beads (ThermoFisher 65602). Beads were washed twice with IP buffer, once with 1 M KCl, once with 0.1 M Na_2CO_3, once with 2 M urea in 10 mM Tris-HCl (pH 8.0), and three times with HEPES pH 8.5 buffer. Proteins were eluted from beads by protease digestion and analyzed by mass spectrometry.

Protein digestion and preparation of mass spectrometry analysis

The samples were digested on-bead in 100μL of 100 mM EPPS, pH 8.5 and at 37°C with 0.5 μg trypsin overnight. The samples were desalted via StageTip, dried via vacuum centrifugation, and reconstituted in 5% acetonitrile, 5% formic acid for LC-MS/MS processing.

Mass spectrometric data collection

Mass spectrometry data were collected using a Exploris 480 mass spectrometer (Thermo Fisher Scientific, San Jose, CA) coupled with a Proxeon 1200 Liquid Chromatograph (Thermo Fisher Scientific). Peptides were separated on a 100 μm inner diameter microcapillary column packed with ∼25 cm of Accucore C18 resin (2.6 μm, 150 Å, Thermo Fisher Scientific). We loaded ∼0.5 μg onto the column.

Peptides were separated using a 60min gradient of 3–22% acetonitrile in 0.125% formic acid with a flow rate of 350 nL/min. The scan sequence began with an Orbitrap MS[1] spectrum with the following parameters: resolution 60,000, scan range 350–1350 Th, automatic gain control (AGC) target "standard", maximum injection time was set to 50ms, RF lens setting 50%, and centroid spectrum data type. We selected the top twenty precursors for MS[2] analysis which consisted of HCD high-energy collision dissociation with the following parameters: resolution 15,000, AGC was set at "standard", maximum injection time "auto", isolation window 1.2 Th, normalized collision energy (NCE) 28, and centroid spectrum data type. In addition, unassigned and singly charged species were excluded from MS[2] analysis and dynamic exclusion was set to 90 s. Each sample was run twice with a CV set of −30/−50/−70 V. A 1 s TopSpeed cycle was used for each CV.

Whole-cell lysis and immunoblotting

Trp63-CreER::LSL-ERTurboID basal cells were expanded in cell culture, treated with 4OHT or vehicle control and lysed in RIPA buffer (50 mM Tris pH 8, 150 mM NaCl, 0.1% SDS, 0.5% sodium deoxycholate, 1% Triton X-100, 1× HALT protease inhibitor). For each sample, 20 μg of protein were immunoblotted and probed with anti-hemagglutinin (HA) antibody (3F10, Roche, 11867423001) on a Licor system.

Flow cytometry

Trp63-CreER::LSL-ERTurboID basal cells were expanded in cell culture, dissociated with trypsin-LE and fixed in 4% paraformaldehyde (PFA). Cells were permeabilized with 0.2% Triton X-100 in DPBS, stained for HA tag (3F10, Roche, 11867423001) in FACS buffer and analyzed on BD FACSAria II system. Data were visualized using FlowJo (Version 10.9, Becton, Dickinson and Company).

Immunofluorescence

In vitro grown airway epithelia from *Sox8-CreER::LSL-ERTurboID* basal cells were fixed directly on the transwell membranes with 4% PFA. Membranes were then excised and stained for SOX8 (Santa Cruz, sc-374445), CCL9 (R and D Systems Inc, AF463), HA tag (3F10, Roche, 11867423001), VCAM1 (429/MVCAM.A, BDBiosciences, 553330) and TNFRSF11B (Biotechne, AF459).

QUANTIFICATION AND STATISTICAL ANALYSIS

Mass spectrometric data processing

Mass spectra were processed using a Comet-based in-house software pipeline, MassPike, licensed by Harvard Medical School, using the following in-software settings. The MS spectra were converted to mzXML using a modified version of ReAdW.exe. Database searching included all entries from the mouse UniProt database (downloaded January 2023), which was concatenated with a reverse database composed of all protein sequences in reversed order. Searches were performed using a 50-ppm precursor ion tolerance. Product ion tolerance was set to 0.03 Th. Oxidation of methionine residues (+15.9949 Da) was set as a variable modification.

Peptide spectral matches (PSMs) were filtered to a 1% FDR.[42,43] PSM filtering was performed using a linear discriminant analysis, while considering the following parameters: XCorr, ΔCn, missed cleavages, peptide length, charge state, and precursor mass accuracy. Peptide-spectral matches were identified, quantified, and collapsed to a 1% FDR and then further collapsed to a final protein-level FDR of 1%. Furthermore, protein assembly was guided by principles of parsimony to produce the smallest set of proteins necessary to account for all observed peptides. Spectral counts were then extracted, and the data were subsequently analyzed. Data are available via ProteomeXchange with identifier PXD068869.

Analysis of secretome data from Kupffer cells and hepatocytes

Proteomics data were filtered to remove frequent contaminants (human keratins, trypsin, media components). To control for differences of loading between samples, raw values for each protein were normalized using the total tandem mass tag (TMT) label signal per sample.

$$normalized\ signal\ =\ raw\ TMT\ signal\ \frac{Mean\ (Total\ TMT\ signal\ across\ all\ samples)}{Total\ TMT\ signal\ in\ sample}$$

Next, low confidence hits were filtered out by removing proteins represented with fewer than 3 peptides. Enrichment scores were calculated for each *ERTurboID* sample over the wild type (no *ERTurboID*) control, using the following formula:

$$enrichment\ score = log\ 2\frac{normalized\ signal\ in\ sample}{normalized\ signal\ in\ wt\ control}.$$

Proteins enriched in all biological replicates and with average enrichment scores greater than or equal to 0.5 across all replicates were selected for further analysis. These proteins represent the Kupffer cell and hepatocyte secretomes (Tables S1 and S2).

Analysis of secretome data from M cells

Proteomics data were filtered to remove frequent contaminants (human keratins, trypsin, media components). The biotinylated BSA spike-in control was used to assess differences in IP efficiency and protein loading between samples. All samples showed similar levels of spike-in, and no further normalization was needed. Next, low confidence hits were filtered out by removing proteins represented with fewer than 5 peptides. Enrichment scores were calculated for each RANKL treated sample over the vehicle control and the secretion inhibitor control, using the following formula:

$$enrichment\ score\ =\ log\ 2\frac{\#\ peptides\ in\ RANKL\ sample+1}{\#\ peptides\ in\ negative\ control+1}.$$

Proteins with enrichment scores greater than or equal to 0.5 over both the vehicle control and the secretion inhibitor control were selected for further analysis. This prefiltered list of enriched proteins was used for correlation analysis between the biological replicates (Figures 3B and 3C). Over 95% of enriched proteins in the luminal set and over 98% of enriched proteins in the basolateral set were represented in both replicates. These subsets of proteins, which are enriched in both biological replicates, represent the M cell secretome. The list comprises 108 proteins: 38 proteins in the luminal secretome and 104 proteins in the basolateral secretome (Tables S8 and S9).

Gene set enrichment analysis

GSEA was performed using Enrichr[38] (Figures 2B, 2C, 2D, S2B, and S2C and Tables S3–S7 and Figures 3D and 3E; Tables S10 and S11), against the relevant databases as stated in Tables S3–S7, S10, and S11.

Subcellular localization annotations

To determine if secretome profiling indeed enriched for secreted proteins, the secretome list was annotated with subcellular localization labels from UniProt using custom R code, made available at OSF at doi:10.17605/OSF.IO/N6CHT. First, a reference list of all

known murine proteins with gene names and subcellular localization information was extracted from the UniProt database. The reference list was then subset to only include proteins with annotations related to the secretory pathway (secreted, endoplasmic, Golgi, exosome/al, cell membrane, plasma membrane, vesicle/vesicular, secretory, endosome/al). The basolateral and luminal secretomes were then queried against the secretory reference list. When a protein detected in our secretome dataset was discovered in the reference list, it was annotated as "localized to the secretory compartment according to UniProt". If a protein was not detected in the reference list, it was annotated as "not secretory or no information available according to UniProt". We then plotted the proportions of secretome proteins with "secretory" and "not secretory" UniProt annotations in Figure 3F.

Comparison of secretomes and transcriptomes from M cells

To determine if secretome profiling has improved sensitivity over single cell transcriptomic data, the M cell secretome was compared to transcriptional data for M cells from our previously published single cell RNA atlas of the murine airway epithelium.[12] To define an M cell transcriptome, the M cell cluster was first subset from the atlas and queried for genes with nonzero expression in at least 5 cells and a total of at least 15 unique molecular identifiers (UMIs), using Seurat (ver. 4). These parameters represent a conservative threshold and were chosen to ensure that even lowly expressed genes are included. The list of genes from transcriptomic data was then compared with the list of proteins from the secretome and plotted as Venn diagrams (Figure 3G; Table S12). Finally, Venn diagrams of the unique and overlapping proteins in the luminal and basolateral compartments were plotted in Figure 3H and listed in Table S13.

Immunofluorescence colocalization

Immunofluorescence data were collected from two independent cell lines with six technical replicates each. Colocalization of staining for M cell markers SOX8 and CCL9 with ERTurboID reporter expression was quantified manually in Fiji and represented as mean and standard deviation in Figure S3.

Figures

Figures were prepared using BioRender.

Raw proteomic data

Raw proteomic counts are made available as Excel files (Tables S14, S15, and S16).

Cell Reports Methods, Volume *6*

Supplemental information

A sensitive method for profiling cell-type-specific secretomes of rare cells from complex tissues

Gergana Shipkovenska, Xuchao Lyu, Manalee Vishnu Surve, Joao A. Paulo, Edward Chen, Vanshika Panchal, Brian Lin, Steven P. Gygi, Wei Wei, Jonathan Z. Long, and Jayaraj Rajagopal

Figure S1. Strategy for inducing cell type-specific ERTurboID. Related to Figure 1.

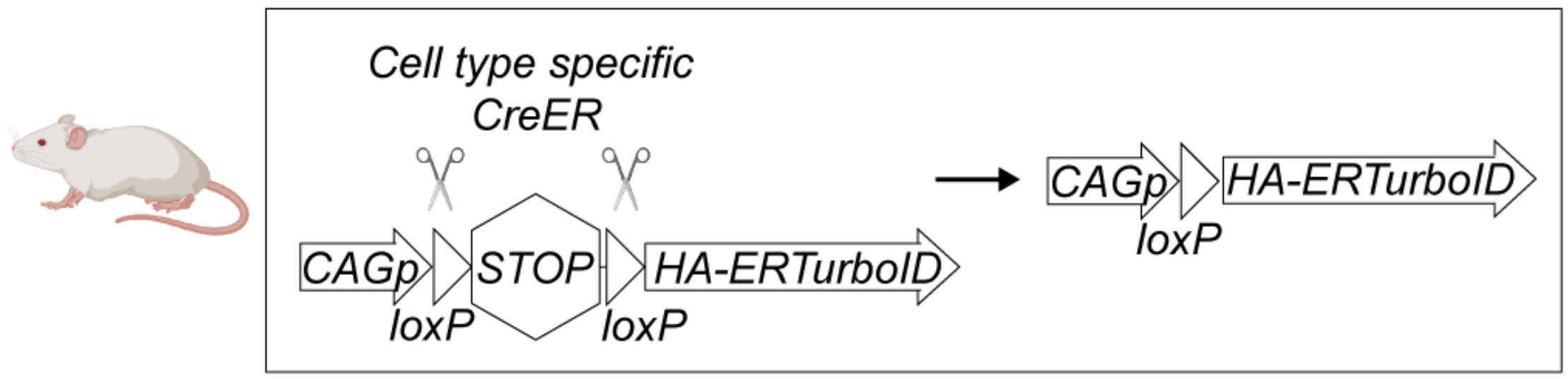

Figure S2. Profiling the secretome of hepatocytes in mice *in vivo*. Related to Figure 2.

A. Schematic of experimental design for profiling the secreted proteome of hepatocytes from intact liver. B. Gene set enrichment analysis of the hepatocyte secretome enriches for terms associated with hepatocyte cell fate. C. GO term analysis of the hepatocyte secretome enriches for terms associated with intracellular compartments involved in protein secretion. P-values were calculated from Fisher exact test. See also Tables S2, S4, S6 and S15.

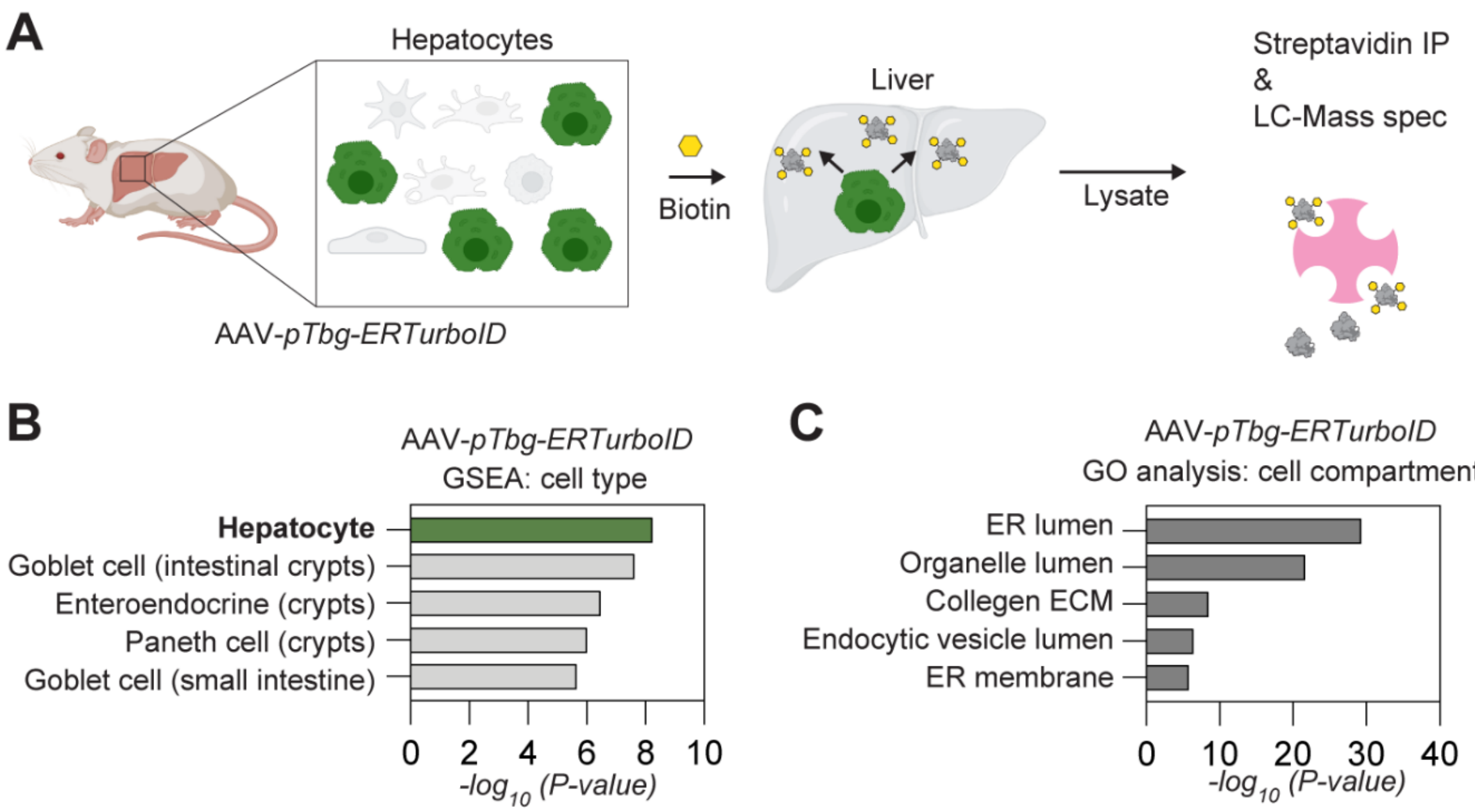

Figure S3. Experimental validation of the *Sox8-CreER* driver. Related to Figure 3.

A. Experimental setup for inducing ERTurboID expression in M cells in air-liquid interface (ALI) cultures. B. Immunofluorescence for M cell markers SOX8 and CCL9 and HA-tagged ERTurboID in fixed ALI cultures of murine airway epithelia. Abbreviations: 4OHT (4 hydroxytamoxifen), RANKL (RANK ligand). C. Quantification of SOX8+ ERTurboID+ double positive cells as proportion of total SOX8 positive cells. Error bars represent standard deviation of two independent cell lines with 6 technical replicates each. D. Quantification of CCL9+ ERTurboID+ double positive cells as proportion of total CCL9 positive cells. Error bars represent standard deviation of two independent cell lines with 6 technical replicates each. E. Quantification of SOX8+ ERTurboID+ or CCL9+ ERTurboID+ double positive cells, as proportion of total ERTurboID positive cells. Error bars represent standard deviation of two independent cell lines with 6 technical replicates each.

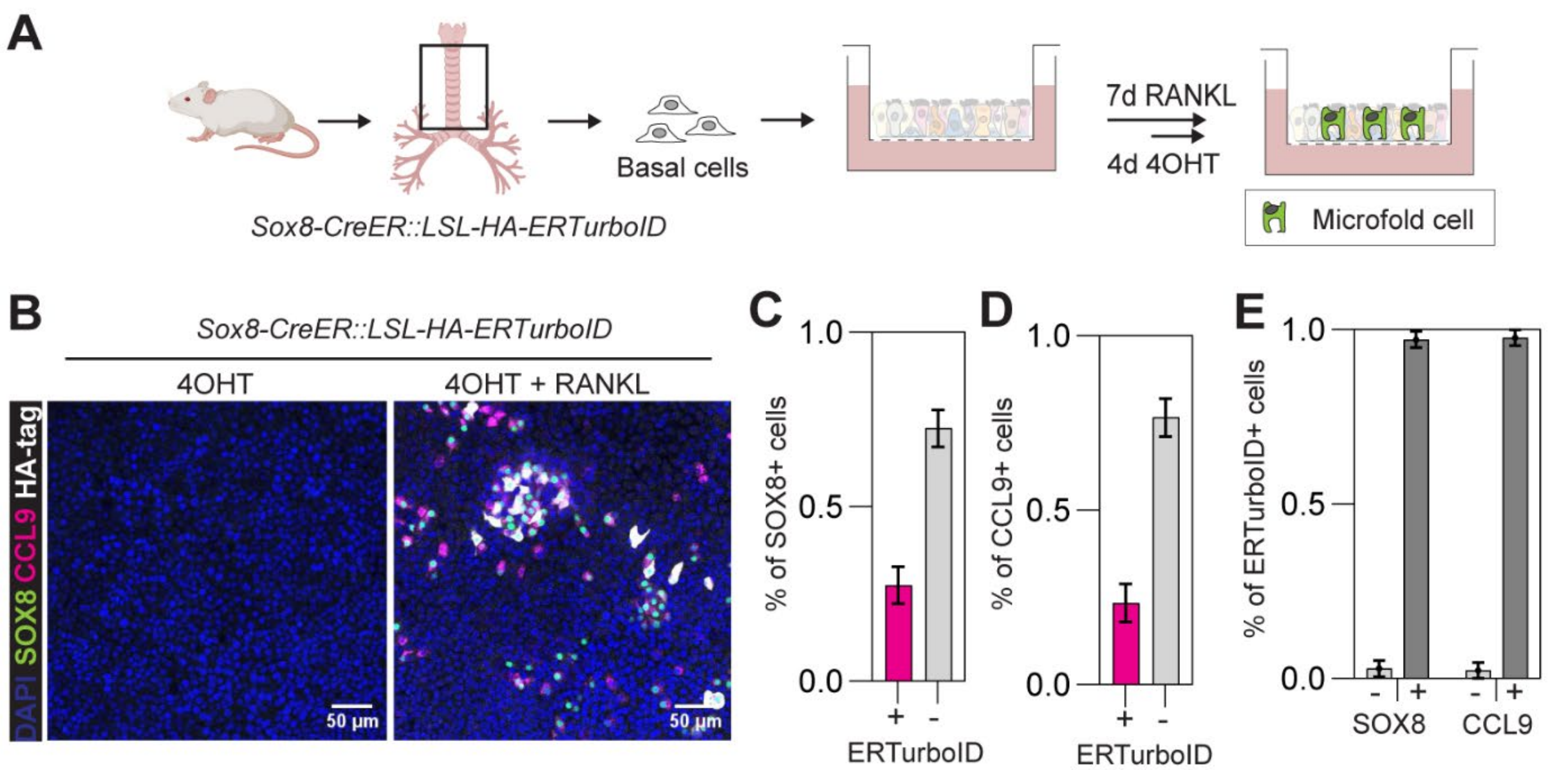

Figure S4. A. Schematic of M cell proteins found in specific compartments of the secretory pathway. B. Immunofluorescence for SOX8, an M cell marker, and TNFRSF11B, which was identified in the M cell secretome. Arrows denote double positive cells. C. Immunofluorescence for SOX8 and VCAM1, which was identified in the M cell secretome. Arrows denote double positive cells. Inset shows magnification of one double positive cell and an orthogonal view along the z-axis to illustrate the luminal-basolateral distribution of VCAM1. Z-sections representing the basolateral and luminal membranes. Related to Figure 4.

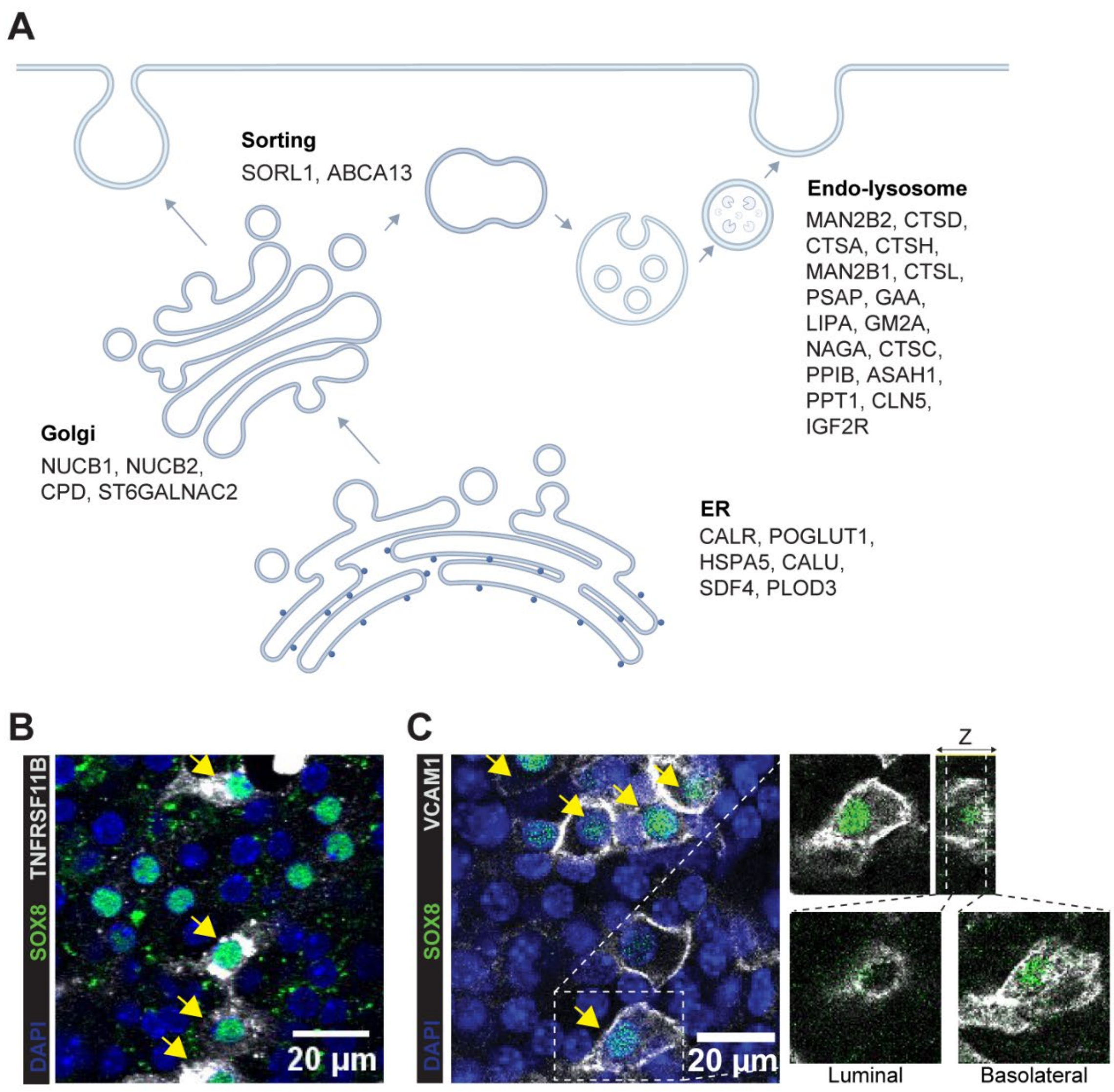

Table S1. List of secreted proteins from Kupffer cells. Related to Figure 2.

Gene	Mean Enrichment Score	Peptides
H1-3	2.068192476	3
Serpina1b	1.565857391	3
TurboID	1.51669736	6
Try4	1.484649371	4
H3c2	1.463571274	5
Cd5l	1.303351435	12
Mia3	1.284448056	14
C1qb	1.185796502	6
Serping1	1.176042236	6
H4c1	1.171030615	5
Apoc1	1.170455665	4
Mrc1	1.100740506	6
Mia2	1.060287106	5
Ganab	1.059353229	7
Ica	1.034277933	9
Apoh	1.028168784	7
Qprt	1.011581575	3
Plg	1.006465476	8
C8g	0.963258798	5
Tpm3	0.961151878	4
H6pd	0.958492919	11
Igkv1-135	0.956383102	4
F5	0.921261736	3
Cfi	0.915050667	4
Cfhr4	0.889943217	5
Ighv3-6	0.886000907	4
Ppia	0.837271137	3
Sdha	0.79476108	3
Ighv8-8	0.761474528	3
Lrp1	0.748487352	14
Aldh6a1	0.708086037	20
Clec4f	0.700801766	4
Rps27a	0.676259129	5
Itih4	0.671665069	14
Ces2e	0.653480864	4
Mpst	0.652614157	3
P4hb	0.642187877	11
Masp2	0.627151401	3
Atp1b1	0.615840486	3
Krt2	0.615290469	5
Atp6v1a	0.613048692	4
Fmo1	0.601157964	5
Dld	0.592919017	5
Pls3	0.561500787	3
Cct2	0.555477939	3
Gapdh	0.551419286	17
Rpl7a	0.546271996	4

Uggt1	0.538522635	9
Atp5pb	0.531088418	5
Nucb1	0.511278665	8
C1ra	0.508431638	5
Ubr4	0.507338881	6

Table S2. List of secreted proteins from hepatocytes. Related to Figure S2.

Gene	Mean Enrichment Score	Peptides
TurboID	4.540224029	25
Creld2	7.094378904	8
Ces3a	5.634971874	22
Ces2e	4.991047056	21
Txn	4.928261864	3
Slco1a1	4.826677035	20
Lypla2	4.766687135	4
Ces1f	4.690017277	35
Tmed10	4.678655188	6
Ces2a	4.660349401	45
Ces1e	4.597166158	15
Cnpy3	4.526766173	6
Ykt6	4.263039871	3
Manf	4.251526506	5
Hspa13	4.012684562	5
Nenf	3.998156244	3
Tmed9	3.988394564	3
Ces2c	3.972517314	17
Creld1	3.900925356	3
Por	3.826866328	12
Prkcsh	3.781182743	14
Slco2b1	3.771868367	5
Pdia3	3.768196921	22
Mesd	3.703087352	12
Pdia4	3.639694069	28
P4hb	3.566127748	43
Cxadr	3.505245909	6
Dnajc3	3.393265269	11
Slc38a3	3.340462264	4
Ero1a	3.307925152	9
Ugt2b36	3.226228089	8
Ces3b	3.193518581	5
Cr1l	3.149322938	3
Hyou1	3.12804866	40
Ero1b	3.09001156	3
Acot2	3.059138254	3
Pcyox1	3.045741912	6
Slco1b2	3.036535014	7
Ppib	2.990278626	3
Ces1d	2.970027545	12
Erp29	2.928201359	4
Lrpap1	2.894345246	11
Ganab	2.888157019	33
Calr	2.870841901	17
Aadac	2.869729728	13
Cnpy4	2.858950646	3
Cyp7b1	2.858768736	3
Mttp	2.853919375	45
Csad	2.810608242	5
Bcap31	2.721963079	7
Ctsh	2.688414569	3

Ces2g	2.668470171	3
Erp44	2.662479779	3
Itgb1	2.649981422	5
Btd	2.623323764	5
Txndc12	2.557570548	3
Ugt2b5	2.518585275	17
H6pd	2.478622775	32
H2-K1	2.467432829	4
Slc9a3r1	2.464274928	3
Selenof	2.451011824	3
Apmap	2.445001551	3
Asah1	2.436091587	3
Lman2	2.431913291	5
Pcyt2	2.420254307	5
Mia3	2.412188929	12
Igfbp2	2.367977519	5
Ces1	2.358982547	10
Coro7	2.342444842	3
Uggt1	2.326399996	27
Bsg	2.233748057	3
Ambp	2.206856355	7
Pofut1	2.133254749	3
Mlec	2.07047889	11
Nucb1	2.008854627	19
F13b	1.967998771	12
Asgr1	1.966187494	6
Col18a1	1.951769465	6
Pebp1	1.868210752	3
Hsd11b1	1.769488656	5
Ctsl	1.763337245	5
Arg1	1.753513148	8
Canx	1.725916678	5
Apoe	1.713968485	10
Timm44	1.64672072	6
Stbd1	1.54164146	3
Lcat	1.536837716	5
Txndc5	1.530036301	4
Dlat	1.453225092	9
Vim	1.44718961	4
Cct8	1.442158416	6
AI182371	1.390925416	10
Ctsd	1.360744343	4
Hrg	1.345929538	12
Man1a1	1.279202792	4
Calu	1.24892885	4
Serpina1b	1.220059127	4
Ca3	1.17151809	11
Klk1b9	1.161944814	6
F2	1.161269476	12
Ugt2b35	1.15065082	10
Cnpy2	1.148333722	3
Alb	1.133199789	59
Hp	1.086918332	7

Apoa4	1.067843856	22
Ces1b	1.065850769	8
Aldh1a1	1.044162996	10
Azgp1	1.029472651	12
Fabp1	1.023229388	3
Prdx6	1.019571399	6
Spcs2	0.960715802	3
Gc	0.916915775	27
Fcn1	0.907971074	3
Serpina3g	0.893332683	11
Abcb11	0.888767941	12
H2-K1	0.879379037	3
Ugt3a2	0.874191579	7
Tkt	0.860572417	3
H4c1	0.839770408	4
Serpina1d	0.837376516	6
Park7	0.805412837	4
Gm20547	0.780716951	17
Fetub	0.747542439	13
Clu	0.715896792	26
Selenbp2	0.695486197	10
Comt	0.688325475	10
Lman1	0.686477808	12
Gclc	0.682851774	4
H2-Q10	0.668616571	6
Ndrg2	0.667199631	4
Serpina1e	0.647953397	3
Serpina3m	0.647772939	35
Gstm1	0.641809707	6
Cfi	0.62987691	14
Apoa1	0.606637175	34
Serpina1a	0.603243317	23
Ctsz	0.599820605	5
C3	0.596452046	70
Kng2	0.587565469	9
F12	0.560299038	4
Serpinf1	0.558713236	7
Tf	0.546796264	74
Cfh	0.542415411	50
Cyb5a	0.540885013	3
C8a	0.537994982	23
Hpx	0.533540971	18
Entpd5	0.525698522	16
C1sa	0.504311858	13

Table S3. Gene Set Enrichment Analysis of the Kupffer cell secretome for cell type-specific markers using the CellMarker 2024 Database (mouse). Related to Figure 2.

term	p-value	q-value	overlap_genes
Kupffer Cell Liver Mouse	0.000004	0.000638	CD5L, MRC1, CLEC4F
Macrophage Peritoneal Cavity Mouse	0.000099	0.003642	CD5L, CLEC4F
Macrophage Bone Marrow Mouse	0.000102	0.003642	C1QB, MRC1, F5
Transit Amplifying (TA) Cell Intestinal Crypt Mouse	0.000342	0.010135	P4HB, RPS27A, PPIA, GAPDH
Macrophage Liver Mouse	0.000591	0.013159	C1QB, CLEC4F

Table S4. Gene Set Enrichment Analysis of hepatocyte secretome for cell type-specific markers using the CellMarker 2024 Database (mouse). Related to Figure S2.

term	p-value	q-value	overlap_genes
Hepatocyte Liver Mouse	5.680601e-09	1.013987e-06	GSTM1, AMBP, ARG1, APOA1, APOE, CYP7B1, ASGR1
Goblet Cell Intestinal Crypt Mouse	2.425794e-08	2.886694e-06	PDIA3, CYB5A, TMED9, GSTM1, MTTP, SELENOF, APOA1, APOA4, MANF, FABP1, LMAN2, BSG, ALDH1A1, TXNDC5
Enteroendocrine Cell Intestinal Crypt Mouse	3.387809e-07	3.023619e-05	FABP1, CYB5A, GSTM1, IGFBP2, MTTP, ALDH1A1, APOA1, APOA4, VIM
Paneth Cell Intestinal Crypt Mouse	9.642755e-07	6.884927e-05	PDIA3, FABP1, GSTM1, BSG, MTTP, ALDH1A1, APOA1, APOA4, P4HB, COMT, NDRG2, TXNDC5
Goblet Cell Small Intestine Mouse	2.216027e-06	1.318536e-04	PDIA3, TMED9, SELENOF, CALR, CRELD2, TXNDC5, MANF

Table S5. Gene Set Enrichment Analysis of Kupffer cell secretome for markers of subcellular compartments using the GO Cellular Component 2025 Database. Related to Figure 2.

term	p-value	q-value	overlap_genes
Endoplasmic Reticulum Lumen (GO:0005788)	5.178072e-08	0.000002	GANAB, H6PD, FMO1, SERPING1, NUCB1, MIA3, P4HB, UGGT1, F5
Intracellular Organelle Lumen (GO:0070013)	5.330122e-08	0.000002	MPST, H6PD, ATP5PB, FMO1, MIA3, SDHA, F5, ALDH6A1, GANAB, SERPING1, NUCB1, P4HB, PPIA, UGGT1
Secretory Granule Lumen (GO:0034774)	1.672427e-05	0.000446	ITIH4, CCT2, APOH, SERPING1, PLG, PPIA, F5
Chylomicron (GO:0042627)	2.359143e-04	0.004718	APOH, APOC1
Vesicle (GO:0031982)	3.222724e-04	0.005156	ATP1B1, RPS27A, PPIA, GAPDH, F5

Table S6. Gene Set Enrichment Analysis of hepatocyte secretome for markers of subcellular compartments using the GO Cellular Component 2025 Database. Related to Figure S2.

term	p-value	q-value	overlap_genes
Endoplasmic Reticulum Lumen (GO:0005788)	5.408981e-30	6.653046e-28	ERO1A, LRPAP1, COL18A1, ERO1B, H6PD, PRKCSH, MTTP, CTSZ, SELENOF, TXNDC12, MIA3, C3, GANAB, APOE, UGGT1, TXNDC5, PDIA3, APOA1, APOA4, CNPY3, MANF, DNAJC3, ERP44, TF, CANX, ERP29, CALU, NUCB1, HYOU1, CALR, P4HB, PPIB, CES1
Intracellular Organelle Lumen (GO:0070013)	2.225699e-22	1.368805e-20	FCN1, ERO1A, COL18A1, ERO1B, ASAH1, H6PD, PRKCSH, MTTP, CTSZ, SELENOF, TXNDC12, MIA3, C3, HPX, GANAB, CTSL, CTSH, APOE, DLAT, CCT8, CTSD, UGGT1, TXNDC5, PDIA3, APOA1, TIMM44, APOA4, CNPY3, MANF, DNAJC3, ERP44, TF, CANX, ACOT2, ERP29, CALU, NUCB1, HYOU1, CALR, P4HB, PPIB, CES1
Collagen-Containing Extracellular Matrix (GO:0062023)	3.423325e-09	1.403563e-07	FCN1, COL18A1, AMBP, F12, SERPINF1, CTSZ, APOA1, APOA4, CLU, HPX, LMAN1, AZGP1, CTSL, CTSH, APOE, CRELD1, CTSD
Endocytic Vesicle Lumen (GO:0071682)	3.707619e-07	1.140093e-05	HPX, CTSL, HYOU1, APOE, CALR
Endoplasmic Reticulum Membrane (GO:0005789)	1.655017e-06	4.071341e-05	BCAP31, ERO1A, CYB5A, ERO1B, TMED9, PCYT2, TMED10, PRKCSH, MIA3, CYP7B1, HSD11B1, ERP44, LMAN1, SPCS2, LMAN2, BSG, CANX, CALU, MLEC, CALR, TKT

Table S7. Gene Set Enrichment Analysis of Kupffer cell secretome for functional pathways using the WikiPathways 2024 Mouse Database. Related to Figure 2.

term	p-value	q-value	overlap_genes
Complement Activation Classical Pathway WP200	0.000879	0.007743	C1QB, C1RA
Statin Pathway WP1	0.001102	0.007743	LRP1, APOC1
Blood Clotting Cascade WP460	0.001223	0.007743	PLG, F5
Amino Acid Metabolism WP662	0.001939	0.009208	MPST, SDHA, DLD
TCA Cycle WP434	0.002938	0.011164	SDHA, DLD

Table S8. Luminally secreted proteins from airway M cells. Related to Figure 3.

Gene	log2 +RANKL/ -RANKL	log2 +RANKL/ +Inhibitor	Unique Peptides	Razor Peptides
Il4i1	3.715226	2.019068	0	161
Mmp7	3.633393	0.729716	0	89
Lama3	3.522197	2.729716	0	68
Lgals3bp	3.522197	3.522197	33	11
Fcgbp	3.133393	3.133393	0	31
Man2b1	2.98864	2.98864	29	0
Man2b2	2.696159	2.696159	10	25
Psap	2.696159	1.696159	0	27
Serpina10	2.696159	2.696159	0	28
Matn2	2.5	2.5	0	29
Ctsh	2.453445	2.453445	0	39
Ecm1	2.453445	1.453445	0	3
Ecm1	2.453445	1.453445	0	68
Lamc2	2.453445	2.453445	7	32
Ctsd	2.437235	1.229716	0	60
Poglut1	2.403677	1.903677	40	0
Fcamr	2.321928	2.321928	0	35
Tgfb2	2.321928	2.321928	6	13
H2-Q4	2.292481	1.292481	0	6
Mfge8	2.289344	0.957861	0	232
Cd14	2.239024	3.642701	75	0
St6galnac2	2.160964	0.868483	39	0
Vcam1	2.160964	2.160964	0	40
Thbs1	2.084963	1.084963	0	18
Icam1	2	1.5	0	19
Ovos	2	2.792481	21	13
Gm20547	1.993964	2.447409	0	102
Sdf4	1.792481	1.292481	5	13
Tnc	1.792481	1.792481	0	12
Tnfrsf11b	1.792481	1.792481	24	0
Hp	1.584963	2.584963	27	0
Clu	1.558894	1.39793	81	129
Pigr	1.549768	1.631517	87	8
Pam	1.292481	1.292481	0	38
Sftpd	0.903677	1.374231	65	0
Mup8	0.792481	2.292481	0	2
Ltf	0.641072	1.53103	139	95
Msln	0.614409	0.710732	0	222

Table S9. Basolaterally secreted proteins from airway M cells. Related to Figure 3.

Gene	log2 +RANKL/ -RANKL	log2 +RANKL/ +Inhibitor	Unique Peptides	Razor Peptides
Nucb1	4.554262	1.094831	0	170
Mfge8	4.518087	4.518087	0	232
App	4.39068	3.39068	0	55
Serpinb1a	4.245927	3.745927	53	65
Aplp2	4.169925	4.169925	0	32
Msln	4.054262	1.163582	0	222
H2-D1	4	3.5	0	64
Calr	3.98864	2.696159	193	0
Vcam1	3.903677	3.903677	0	40
Il4i1	3.877444	2.292481	0	161
Lama3	3.836213	3.836213	0	68
H2-K1	3.753897	3.253897	0	58
Bpifb1	3.745927	1.049768	148	0
Poglut1	3.70044	2.70044	40	0
Man2b2	3.696159	3.696159	10	25
Mmp7	3.696159	1.535195	0	89
Chadl	3.642701	3.642701	8	15
Gm20547	3.642701	3.642701	0	102
Cln5	3.633393	3.633393	26	0
Sorl1	3.633393	3.633393	29	0
Igf2r	3.564642	3.564642	33	0
Ptprk	3.522197	3.522197	0	21
Ctsd	3.511184	2.011184	0	60
Dsg2	3.453445	3.453445	92	47
Met	3.447409	3.447409	0	26
Lgals3bp	3.39068	3.39068	33	11
Plxnb2	3.39068	3.39068	52	0
Tnfrsf11b	3.39068	3.39068	24	0
Cd14	3.377444	3.377444	75	0
Matn2	3.377444	3.377444	0	29
Spint1	3.321928	3.321928	23	0
Ltf	3.253897	1.85022	139	95
Ptprz1	3.245927	3.245927	0	17
Pigr	3.20044	3.20044	87	8
Serpina10	3.169925	3.169925	0	28
Ctsa	3.160964	3.160964	0	27
Ctsh	3.084963	3.084963	0	39
Lamc2	3.084963	3.084963	7	32
Fcgbp	3.064642	3.064642	0	31
Man2b1	3.064642	3.064642	29	0
H2-Q4	3	2.5	0	6
Ldlr	3	3	0	17
Fat1	2.98864	2.98864	0	20
Lamb3	2.953445	2.953445	29	0
Nucb2	2.903677	0.903677	0	75
Psap	2.877444	2.877444	0	27
Cd74	2.807355	2.807355	1	34
Ctsl	2.807355	1.514874	1	34
L1cam	2.807355	2.807355	0	14

Sftpd	2.792481	2.792481	65	0
Apoa4	2.745927	2.745927	13	0
Fcamr	2.696159	2.196159	0	35
Gaa	2.696159	2.696159	3	9
Clu	2.685779	2.554262	81	129
Abca13	2.660964	2.660964	0	12
Clstn1	2.660964	2.660964	0	11
Cpd	2.660964	2.660964	13	0
Serpinb11	2.584963	2.584963	34	18
Agrn	2.564642	2.564642	0	12
Lipa	2.564642	2.564642	11	0
Hspa5	2.522197	1.568752	238	37
Calu	2.453445	1.453445	12	0
Calu	2.453445	1.453445	0	38
Gm2a	2.453445	2.453445	17	0
Itga2	2.453445	2.453445	9	0
Ces2f	2.321928	2.321928	0	9
Lsr	2.321928	2.321928	0	11
Sdf4	2.321928	2.321928	5	13
Calcb	2.292481	1.792481	0	14
Plxnb1	2.292481	2.292481	12	3
Tgfb2	2.292481	2.292481	6	13
Ctsc	2.160964	2.160964	0	10
Dag1	2.160964	2.160964	8	0
Naga	2.160964	2.160964	5	8
Nenf	2.160964	1.368483	15	0
Sema3d	2.160964	2.160964	7	0
Thbs1	2.160964	2.160964	0	18
Plod3	2.084963	2.084963	4	3
Ccl20	2	2	0	20
Fetub	2	2	7	14
Glycam1	2	2	0	9
Icam1	2	2	0	19
Ppib	2	1.5	59	0
Rnaset2b	2	2	0	16
Tnc	2	2	0	12
Hp	1.953445	1.453445	27	0
Lbp	1.953445	1.953445	1	42
Cp	1.903677	1.611196	0	303
Asah1	1.792481	1.792481	8	0
Dsc2	1.792481	1.792481	0	21
Env1	1.792481	1.792481	11	0
Grn	1.792481	1.792481	0	6
Pam	1.792481	1.792481	0	38
Ppt1	1.792481	1.792481	4	1
Prnp	1.792481	1.292481	15	0
Sema3a	1.792481	1.792481	0	5
UPF0764	1.792481	1.792481	9	0
Tf/Trf	1.77216	1.111196	11	31
Ecm1	1.660964	0.868483	0	68
Pon1	1.660964	1.660964	15	67
Ecm1	1.453445	0.660964	0	3
B2m	1.403677	1.403677	22	0

Lgi2	1.292481	2.292481	0	40
Sulf2	1.292481	1.792481	6	0
Dsg1b	1.216056	2.043731	0	26
Sftpa1	0.868483	2.160964	0	49
Cdh1	0.696159	2.696159	0	27

Table S10. Gene Set Enrichment Analysis of the M cell secretome for markers of cell type using the PanglaoDB Augmented 2021 Database. Related to Figure 3.

term	p value	q value	overlap_genes
Microfold Cells	2.71E-08	3.15E-06	GRN, SPINT1, LAMB3, CCL20, CTSH, CD14, MFGE8, CTSD, ICAM1
Pulmonary Alveolar Type II Cells	6.99E-07	4.05E-05	PIGR, SPINT1, LAMB3, SFTPD, CTSH, CD14, SFTPA1, LTF
Epithelial Cells	2.83E-06	1.09E-04	PIGR, SPINT1, LAMB3, CDH1, CCL20, ITGA2, LAMC2, ICAM1, LTF
Ductal Cells	7.45E-06	2.16E-04	PIGR, MMP7, LAMB3, CDH1, LAMA3, CTSH, LAMC2
Spermatozoa	2.64E-05	6.12E-04	PIGR, CD74, ASAH1, CD14, CP, CLN5

Table S11. Gene Set Enrichment Analysis of the M cell secretome for markers of subcellular compartments using the GO Cellular Component 2025 Database. Related to Figure 3.

term	p value	q value	genes
Collagen-Containing Extracellular Matrix (GO:0062023)	1.69E-14	1.18E-12	LGALS3BP, TGFB2, ECM1, LAMB3, PLOD3, LAMC2, APOA4, L1CAM, CLU, THBS1, ICAM1, CTSL, CHADL, DAG1, CTSH, MFGE8, AGRN, CTSD, CTSC, MATN2
Endoplasmic Reticulum Lumen (GO:0005788)	1.92E-14	1.18E-12	APP, POGLUT1, HSPA5, APLP2, SERPINA10, APOA4, MSLN, CP, THBS1, TF, DAG1, CALU, NUCB1, CALR, PPIB, MFGE8, B2M, CTSC
Lytic Vacuole (GO:0000323)	1.65E-13	6.76E-12	CTSA, APP, CD74, GRN, ASAH1, GAA, SFTPD, CLN5, MAN2B2, CTSL, PPT1, CTSH, MAN2B1, CTSD, LDLR, CTSC
Lysosomal Lumen (GO:0043202)	1.18E-12	3.63E-11	CTSA, CD74, ASAH1, GM2A, CTSL, MAN2B2, GAA, PPT1, MAN2B1, AGRN, CTSD
Intracellular Organelle Lumen (GO:0070013)	1.86E-12	4.58E-11	APP, ASAH1, POGLUT1, SERPINA10, MSLN, THBS1, CTSL, DAG1, CTSH, SDF4, B2M, CTSD, LDLR, CTSC, HSPA5, APLP2, APOA4, CP, TF, CALU, NUCB1, CALR, PPIB, MFGE8, AGRN, LTF
Vacuolar Lumen (GO:0005775)	4.55E-12	9.33E-11	CTSA, CD74, GRN, ASAH1, GAA, GM2A, MAN2B2, CTSL, PPT1, MAN2B1, AGRN, CTSD, CTSC
Lysosome (GO:0005764)	7.39E-12	1.30E-10	CTSA, APP, CD74, GRN, ASAH1, GAA, SFTPD, CP, CLN5, GM2A, MAN2B2, CTSL, PPT1, CTSH, MAN2B1, AGRN, B2M, CTSD, LDLR, CTSC
Secretory Granule Lumen (GO:0034774)	1.42E-11	2.18E-10	CTSA, LGALS3BP, APP, TGFB2, ECM1, GRN, CLU, THBS1, TF, GM2A, CTSH, MAN2B1, B2M, CTSD, CTSC, LTF
Late Endosome (GO:0005770)	3.30E-08	4.52E-07	CD74, TF, GRN, CTSL, SFTPD, SDF4, SFTPA1, B2M, LDLR, SORL1, IGF2R
High-Density Lipoprotein Particle (GO:0034364)	2.93E-06	3.60E-05	APP, PON1, APOA4, CLU

Table S12. Proteins enriched in secretome but absent from scRNA data. Related to Figure 3.

Env1
Agrn
Lgi2
Tgfb2
Gm20547
Matn2
Sema3a
Chadl
Tnc
Sulf2
Hp
Apoa4
Glycam1
Fcamr
Lipa
Tf/Trf
Dsg1b
Mup8
Fetub
Igf2r
UPF0764
Ecm1
Mmp7
Ces2f
Lama3
Man2b2
Ovos
Lamb3
Itga2

Table S13. Proteins enriched in luminal secretome, basolateral secretome, or both. Related to Figure 3.

Basolateral and Luminal	34	Ltf, Fcgbp, Fcamr, Sftpd, Icam1, Tnc, Msln, Tgfb2, Lgals3bp, Poglut1, Lama3, Pigr, Sdf4, Tnfrsf11b, Cd14, H2-Q4, Ctsh, Ecm1, Lamc2, Serpina10, Matn2, Hp, Thbs1, Man2b2, Pam, Vcam1, Il4i1, Gm20547, Psap, Ctsd, Mfge8, Clu, Man2b1, Mmp7
Luminal only	3	Mup8, St6galnac2, Ovos
Basolateral only	71	Serpinb1a, Ppt1, Dag1, Ces2f, Rnaset2b, Agrn, Serpinb11, Plxnb2, Fat1, Hspa5, App, Igf2r, Dsc2, Cd74, H2-K1, Glycam1, Pon1, Tf/Trf, Calu, Asah1, Abca13, Ctsl, Cpd, Env1, Calr, Sorl1, Cp, Dsg2, Ptprk, B2m, Lsr, Plxnb1, Cln5, Ptprz1, Ppib, Lgi2, Nucb2, Cdh1, Clstn1, Sftpa1, Ldlr, Chadl, Plod3, Apoa4, Bpifb1, Sulf2, Sema3a, Naga, Ctsa, Lamb3, Nucb1, Ccl20, Gm2a, Met, Grn, Sema3d, Lbp, H2-D1, L1cam, Lipa, Itga2, Spint1, Calcb, Nenf, Aplp2, Ctsc, Prnp, Fetub, Gaa, UPF0764, Dsg1b
